# Design Considerations for the Integrated Delivery of Cognitive Behavioral Therapy for Depression: User-Centered Design Study

**DOI:** 10.2196/15972

**Published:** 2020-09-03

**Authors:** Katarzyna Stawarz, Chris Preist, Debbie Tallon, Nicola Wiles, David Kessler, Katrina Turner, Roz Shafran, David Coyle

**Affiliations:** 1 School of Computer Science and Informatics Cardiff University Cardiff United Kingdom; 2 Department of Computer Science University of Bristol Bristol United Kingdom; 3 Bristol Medical School University of Bristol Bristol United Kingdom; 4 National Institute for Health Research Bristol Biomedical Research Centre at University Hospitals Bristol and Weston NHS Foundation Trust and the University of Bristol Bristol United Kingdom; 5 The National Institute for Health Research Applied Research Collaboration West Bristol United Kingdom; 6 UCL Great Ormond Street Institute of Child Health University College London London United Kingdom; 7 School of Computer Science University College Dublin Dublin Ireland

**Keywords:** cognitive behavioral therapy, depression, mental health, blended therapy, integrated therapy, user-centered design, qualitative research

## Abstract

**Background:**

Adherence to computerized cognitive behavioral therapy (cCBT) programs in real-world settings can be poor, and in the absence of therapist support, effects are modest and short term. Moreover, because cCBT systems tend toward limited support and thus low-intensity treatment, they are typically most appropriate for people experiencing mild to moderate mental health difficulties. Blended therapy, that is, combining direct therapist contact with cCBT or psychoeducational materials, has been identified as one possible approach to address these limitations and widen access to individual CBT for depression. Building on the initial success of blended therapy, we explore an integrated approach that seeks to seamlessly combine face-to-face contact, electronic contact, and between-session activities. Integration also considers how the technology can support therapists’ workflow and integrate with broader health care systems. The ultimate aim is to provide a structure within which therapists can deliver high-intensity treatments, while also greatly reducing face-to-face contact.

**Objective:**

The research aimed to explore patients’ and therapists’ views on using a system for the delivery of individual treatment for depression that integrates face-to-face therapist contact with access to online resources and with synchronous online therapy sessions that allow collaborative exercises, and to establish design requirements and thus key design considerations for integrated systems that more seamlessly combine different modes of communication.

**Methods:**

We conducted a series of four user-centered design studies. This included four design workshops and seven prototype testing sessions with 18 people who had received CBT for depression in the past, and 11 qualitative interviews and three role-play sessions with 12 CBT therapists experienced in the treatment of depression. Studies took place between July and December 2017 in Bristol, United Kingdom.

**Results:**

Workshops and prototyping sessions with people who had received CBT identified three important requirements for integrated platforms delivering CBT therapy for depression as follows: (1) features that help to overcome depression-related barriers, (2) features that support engagement, and (3) features that reinforce learning and support the development of new skills. Research with therapists highlighted the importance of the therapist and client working together, the impact of technology on therapists’ workflow and workload, challenges and opportunities related to the use of online resources, and the potential of technology to support patient engagement. We use these findings to inform 12 design considerations for developing integrated therapy systems.

**Conclusions:**

To meet clients’ and therapists’ needs, integrated systems need to help retain the personal connection, support both therapist- and patient-led activities, and provide access to materials and the ability to monitor progress. However, developers of such systems should be mindful of their capacity to disrupt current work practices and increase therapists’ workload. Future research should evaluate the impact of integrated systems on patients and therapists in a real-world context.

## Introduction

### Background

Cognitive behavioral therapy (CBT) is an effective treatment for depression [1]. To make it more accessible and widely available at a lower cost, computerized CBT (cCBT) interventions have been developed. They allow patients to complete a set of modules in their own time, giving them control over their own therapy (eg, MoodGYM [2] and SilverCloud [3]). Some cCBT packages have been endorsed by the National Institute for Health and Clinical Excellence (NICE) as part of the stepped care pathway in the treatment of depression in the United Kingdom, mostly to provide low-intensity treatment [4]. However, adherence to cCBT is often poor owing to low acceptability and a lack of therapist involvement [5] and, as a result, effects are modest and short term [6]. Moreover, cCBT is often inflexible and does not allow identification of conditional beliefs or detailed formulations [7] that are crucial elements of CBT and important for those with more severe and chronic depression [8], and for long-term outcomes [9].

“High intensity” and “low intensity” are terms used in the United Kingdom to distinguish two types of mental health support. Low-intensity interventions are generally brief, with a smaller number of sessions, usually about six. These can be delivered via phone or in a group setting, may use a health technology such as guided self-help, and are typically delivered by a paraprofessional. cCBT with some therapist support is offered in UK Improving Access to Psychological Therapies (IAPT) as one of a suite of low-intensity interventions for less severe illnesses. High-intensity treatment is usually delivered individually and face-to-face by a more expert therapist over more and longer sessions (characteristically 12 1-hour sessions). Current UK evidence does not support the idea that cCBT alone can be an alternative to high-intensity CBT [10]. Nevertheless, because of its structured approach, CBT is particularly suited to the integration of computer and mobile technology with a therapist-led treatment. Exercises that take place outside the psychotherapeutic sessions are an important part of CBT, and adherence to these can increase effectiveness [6,11]. Enabling patients to complete exercises, such as worksheets online, as opposed to doing them on paper, may improve adherence and engagement [12], and accessing them on mobile devices may enable discreet and convenient ways of completing them [13]. Working online with specially adapted interactive materials can be supported by timely reminders and wider use of digital media. Moreover, previous research has shown that real-time delivery of CBT using instant messaging is acceptable and effective [14-17], and there is evidence that cCBT with additional guidance from a therapist can be as effective as face-to-face therapy [18-20] and may save clinician time without reducing effectiveness [21].

In recent years, blended CBT has emerged as a promising alternative to cCBT. Blended systems combine online components with direct contact with a therapist [22-26], and initial evidence suggests that this approach is acceptable to patients as a way of receiving therapy and engaging with treatment [22,26]. Literature on blended therapy describes different combinations of online components and therapist contact, including the use of existing cCBT systems with limited feedback from the therapist [23], face-to-face therapy with additional access to online resources [22], or a combination of therapy sessions with online modules and mood tracking [24]. This paper not only draws on the lessons of blended therapy, but also takes an additional step, with focus on the development of more fully *integrated systems* to support high-intensity CBT. Rather than combining face-to-face contact with existing online support (eg, cCBT systems), an integrated approach focuses on the ground up development of platforms that more seamlessly integrate face-to-face contact, electronic contact, online sessions and collaboration, and between-session activities. Collaborative activities, which the therapist and patient complete together during online sessions, and between-session activities, which patients complete on their own, are complementary, as are face-to-face and electronic contact. Integration not only includes providing different options for how the therapist and patient work together, but also considers how the system supports the therapist’s workflow and how the system could be integrated with current practices and broader health care systems. The overall aim is to support patient engagement and provide a structure within which therapists can deliver high-intensity treatment, while also greatly reducing face-to-face contact.

### Objectives

The overall objective of this paper is to provide design guidelines for integrated platforms that support high-intensity CBT. A recent systematic review found that digital mental health technologies that show potential in randomized controlled trials (RCTs) are often less successful when deployed in real-world settings, that is, as implemented (disseminated) outside of research settings [27]. The authors conclude that this issue can be partially addressed through the collection and reporting of implementation data on an ongoing basis. However, it can also be addressed pre-RCT through a process of user-centered design [28]. Central to user-centered design is strong evidence that the long-term success of digital systems is greatly improved by actively involving potential users of a future technology throughout the design lifecycle of that technology. User-centered design recognizes that it is not possible to fully state the requirements of a novel digital system at the outset of the design process. Instead it emphasizes the need for requirements to be developed and refined on an iterative basis through active involvement of representative users. This involvement can take a number of forms, including design workshops, where potential functionalities and problems are mutually explored [29], and the evaluation and critique of early prototype systems with users. Through these approaches, the resulting technology is more able to incorporate the needs, values, and lived experiences of potential users. The value of user-centered design in developing digital health interventions is increasingly recognized [30-32], and its use is becoming more commonplace [33,34].

The research described in this paper involved the first stage of the INTERACT project (a large program of research that brings together a multidisciplinary team to develop and evaluate a platform for delivering integrated therapy for depression). The details of a longitudinal pilot study evaluating the near-final version of the platform have been provided [35]. The project will ultimately result in a large-scale multicenter RCT of the INTERACT platform. In this paper, the overarching goal is to explore how best to design an online platform that enables close integration of direct therapist contact with access to online resources. Addressing this goal resulted in design recommendations that were directly relevant to the INTERACT platform [35]. However, these design recommendations also provide guidance that can be generalized to support the design of other integrated systems. This generalizable guidance is the core contribution of this paper.

## Methods

### Design

**Figure 1:**
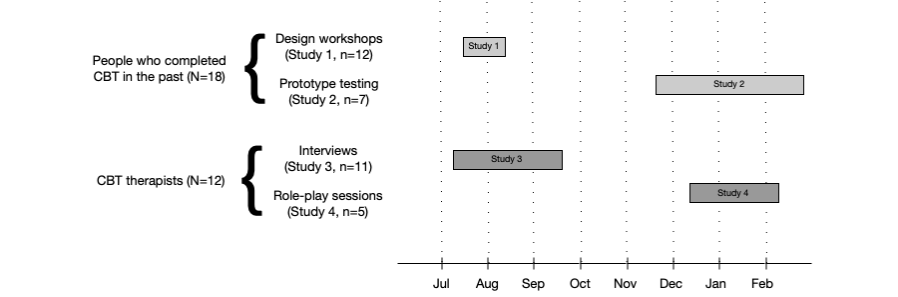
Study flow and number of participants. The period between the studies was dedicated to the analysis of Study 1 and 3 results, and identifying design requirements that informed the prototypes used in Studies 2 and 4. The research took place in 2017.

The objectives in the paper are addressed through a series of user-centered design studies with patients and therapists. User-centered design methods differ from traditional qualitative approaches in that potential users are actively encouraged to make suggestions with regard to the potential design and functionality of the system. In total, we conducted four studies. Our first two studies (1 and 3) focused on identifying general high-level requirements for an online platform for delivering integrated therapy for depression. Our later studies (2 and 4) addressed these requirements in greater detail, making use of concrete system prototypes to develop detailed design guidelines. Two studies were conducted with people who received therapy in the past, which allowed us to understand their needs (Study 1, design workshops) and test patient-facing components of the platform (Study 2, prototype testing sessions). Two studies were also conducted with therapists to gather the initial requirements (Study 3, interviews) and to validate and explore them in greater depth (Study 4, therapy session role plays). Figure 1 presents the order and length of the studies and how they related to each other. Each study has been described in more detail in the following sections.

The prototypes used in later studies (Studies 2 and 4) were informed by the requirements gathered during workshops and interviews (Studies 1 and 3, respectively). The requirements were discussed among the research team and reported to the development team to identify what type of functionality may be needed, how it may work, and what types of activities will keep people engaged with the treatment. While it is not possible (owing to space constraints) to provide full details of each design decision, it is helpful to provide two illustrative examples of this process. During workshops in Study 1, participants expressed strong preferences with regard to tracking their progress. They did not want to see how much work they still had left to complete, but preferred to see how far they have come. The prototype tested in Study 3 reflected this finding and explicitly showed a record of past sessions and worksheets shared by the therapist. Similarly, during therapist interviews (Study 2) when discussing communication with patients between therapy sessions, therapists expressed concerns over increased workload and patients sending large amounts of messages. To address this concern, the prototype used in Study 4 included a platform inbox that asked patients to choose a specific topic for their message (eg, reschedule a session and worksheet query) to keep it focused and reduce between-session communication

The research was approved by the National Health Service (NHS) Ethics Committee (Integrated Research Application System Study ID: 221433) and had Health Research Authority approval.

### Participants

#### People Who Received CBT in the Past

We recruited people who received CBT in the past through local IAPT services and people who had participated in earlier RCTs conducted by members of the research team [9]. The latter group had received one-to-one CBT as part of the trial for treatment-resistant depression and had consented to being contacted about future research.

Individuals eligible to take part were those aged 18 years or older, who had a history of depression and had received CBT for depression in the past. Excluded were those who were currently receiving treatment from a psychiatrist for depression; had a history of bipolar disorder, schizophrenia, personality disorder, or substance misuse/alcohol addiction (in the past year); or reported during the screening call that they were not feeling well enough to attend sessions. All participants were given an option to attend the workshops (Study 1) and/or participate in prototype testing sessions (Study 2).

In total, we recruited 18 participants. Among these, 12 attended the workshops (Study 1) and seven attended the prototype testing sessions (Study 2); one person participated in both activities. The mean age of the participants was 48.5 years (SD 13.4 years; range 22-72 years), and the majority (13/18, 72%) were women; eight had participated in previous trials and 10 were recruited from local IAPT services. Only four had experience with cCBT. Full background details are provided in Multimedia Appendix 1.

#### CBT Therapists

We recruited therapists by contacting those who previously had worked with the research team on an earlier trial [9] and contacting clinical/service leads of local IAPT services to ask them for their support in promoting the study to therapists providing high-intensity CBT within their service (ie, treatment that is delivered to people with medium or severe depression over a longer time period, predominantly face-to-face, and that focuses on both behavioral and cognitive aspects of therapy). We telephoned potentially interested therapists to provide more details or arranged short sessions to describe the study to a group of potential participants at their service. In total, we recruited 12 therapists. Among these, 11 attended the individual interviews (Study 3) and five attended the role-play sessions (Study 4); four attended both. The mean age of the therapists was 43 years (SD 8.8 years; range 30-57 years), and the majority (13/18, 72%) were women. They were all white people. On average, they had worked as a CBT therapist for 8.1 years (SD 5.0 years; range 3-20 years). Among them, 10 worked for the NHS, one for a private practice, and one for both. Further details are provided in Multimedia Appendix 2.

### Study 1: Design Workshops With People Who Received CBT in the Past

#### Materials

**Figure 2:**
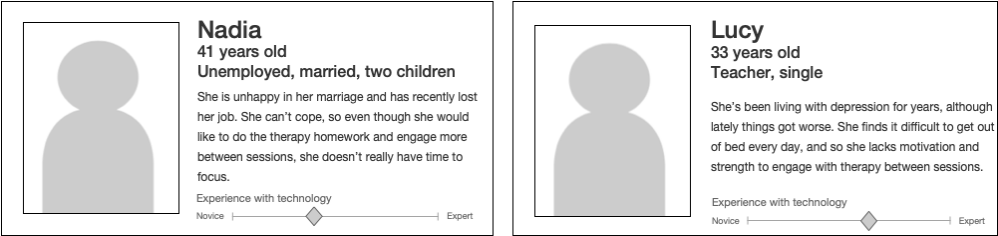
Example patient personas.

We created four short patient personas (see Figure 2 for examples) to serve as prompts during workshops. User personas are part of the user-centered design process [36-38]. They are a way to represent typical users of a computer system and help to empathize with target users and understand their needs. Our personas represented people with depression to illustrate varying circumstances and reasons for treatment (they were aged 19-48 years; two were women; one had comorbid anxiety and one was dealing with grief), as well as additional information about their technical skills. Further details are provided in Multimedia Appendix 3. They were created in collaboration with clinicians on the research team (DK and RS) and coauthors who worked on depression trials in the past (NW, DK, KT, and DT) to represent a range of potential target patients who could benefit from the system we were developing. We used them to make it easier for participants to draw from their own experiences of CBT without the need to explicitly describe their own situation and to help them reflect on how CBT could be improved for others.

#### Procedures

In Study 1, we conducted four design workshops. The first two took place in July 2017, and the discussion focused on barriers of access to cCBT and CBT more broadly, and how technology could widen access. The final two workshops took place in August 2017. They focused on engagement with therapy and therapeutic materials, identifying barriers to engagement and exploring how technology could help to overcome them.

The workshops were facilitated by two researchers (KS and DT). Each workshop lasted 2 hours and was attended by three to five people. All workshops started with a short description of a potential integrated platform for delivering CBT and planned activities for the session. The attendees had the opportunity to ask questions, and then, written informed consent was obtained. Participants were asked to complete a short questionnaire covering sociodemographic details, information about their history of depression, and the treatment they received in the past. After the introductions and a warm-up activity, attendees were divided into two groups. Groups discussed the topics specific for each workshop (ie, access to cCBT and engagement), how they might affect the user personas, and how technology could help to overcome barriers and support engagement.

At the end of each workshop, each attendee received a £20 gift voucher. With participant consent, all workshops were audio recorded.

### Study 2: Prototype Testing With People Who Received CBT for Depression in the Past

To validate the requirements gathered as part of Study 1, we conducted a series of prototype testing sessions. Known as usability testing sessions [39], their aim is to identify issues and areas for improvement during real-life usage scenarios. Evidence suggests that small numbers of participants (n=5-15) are sufficient to identify the key user-centered issues in a prototype [39,40].

#### Materials

Based on the results of Study 1 and prior literature, we built a functional prototype of an online platform for delivering integrated therapy for depression. The prototype included a homepage that showed the time of the next session, homework tasks, and a list showing worksheets that have been shared with the patient by the therapist; a personal profile page with a field for therapy goals; a library of resources listing several psychoeducational resources; and an “online session page” with an instant messenger enabling synchronous communication with the therapist and collaborative worksheet editing. Screenshots of the prototype are presented in Multimedia Appendix 4.

#### Procedures

Prototype testing sessions were conducted one-to-one with a researcher and each lasted approximately 60 minutes. Written informed consent was obtained from all participants. Those who had not attended earlier design workshops were also asked to complete a brief questionnaire about their background.

To gain insights into participants' thinking, sessions involved a think aloud protocol [39], that is, participants were given specific tasks to complete (eg, complete worksheets, send a message to a therapist, and participate in an online therapy session) and were asked to describe what they were thinking while they were doing it to encourage them to comment on the experience. To provide context for the tasks, participants were given patient cards based on the user personas from Study 1, which provided information about recent events and their worries (see Multimedia Appendix 4 for more details). They were asked to complete the tasks on behalf of these patients rather than provide their own information to help them generalize their experiences. Each session started with a practice task to familiarize participants with thinking aloud when using a prototype. All sessions were audio-recorded with consent. Each participant received a £10 gift voucher for their participation.

### Study 3: Qualitative Interviews With Therapists

#### Procedures

Therapists were interviewed at their workplace or at the university; one therapist was interviewed at home. The interviews lasted 45 to 60 minutes and were conducted by the first author. Each interview started with questions about the therapist’s current approach to delivering CBT for depression, including their use of worksheets and other materials, client engagement, and their use of technology. Thereafter, the researcher described a potential integrated system to elicit feedback and gather further views regarding using technology for delivering CBT. Finally, the researcher showed paper prototypes [41] illustrating some of the features that could be available in an integrated system to elicit further feedback and help improve the design. The paper prototypes (Multimedia Appendix 5) were based on the literature on blended therapy systems, existing services (eg, Ieso [42]), the results of Studies 1 and 2, and insights from the therapists on the research team. They also served as a starting point for a discussion about the use of technology to manage workload, keep track of clients and their needs, and deal with risk. The interviews were audio-recorded and transcribed verbatim by an external transcription service.

For their participation, each therapist received £27. The rate was worked out on the basis of their standard hourly rate. If therapists were interviewed during their usual working hours, the payment was made to their employer, but if they were interviewed during nonworking hours, the payment was made to them directly.

### Study 4: Platform Role-Play Sessions With Therapists

To evaluate the prototype of the platform with therapists, we conducted therapy role plays with CBT therapists. Role plays have been successfully used to design therapeutic systems and are well suited to the mental health care setting [43]. This format enabled therapists to better understand how delivering integrated therapy could work in practice and helped to elicit therapists’ tacit knowledge of therapy interactions.

#### Materials

Each therapist used a separate laptop during the session. To support the role plays, we used the same prototype used in Study 2 and set up demonstration accounts with therapist and patient information already added, including homework tasks, completed worksheets, and therapy goals. Based on the personas from Study 1, we provided patient cards and patient scenarios. Participants role playing “patients” received a more detailed scenario card that summarized events that had taken place since their last therapy session and suggested topics to cover during the role play. The scenarios were informed by the types of patients described by therapists in Study 3 and reflected the main components of CBT [44]. They were approved by clinicians (DK and RS) on the research team after running a test role-play session during which one researcher (DT) acted as a patient and another researcher (DK) acted as a therapist. All study materials, including screenshots of the platform and patient scenarios, are presented in Multimedia Appendix 6.

#### Procedures

We conducted three role-play sessions (two sessions with pairs of therapists and one session with a therapist paired up with a researcher [DT] who acted as a patient). Each session lasted 90 minutes and was facilitated by the first author. After obtaining written informed consent, therapists were asked to complete a brief questionnaire to provide information about their sociodemographic characteristics, professional background, and experience.

To familiarize them with the prototype, therapists were walked through it first. Thereafter, they were given patient cards and specific scenarios in order to role play a session. The therapist role playing a “therapist” was given a simple patient card and was able to look up patient information in the system. The therapist role playing a “patient” was given a patient card and a scenario card. Therapists were free to run the session however they wanted with minimum input from the researchers who were present to take notes. They were able to pause the role play at any time to comment on the system, ask for clarifications, and offer suggestions for improvements. Role plays were followed by a discussion about therapists’ experience of using the prototype, their views on delivering therapy this way, and their views on how a system like this could fit into their current practice. The sessions were audio-recorded with consent. Each therapist was paid £41 for their time; this was based on their hourly rate. If the session was conducted during the therapist’s work time, we paid their employer, but if it was conducted during nonwork time, we paid the therapist directly.

### Data Analysis

Notes from the workshops (Study 1) were copied onto sticky notes, and together with sticky notes generated by the participants, they were used in affinity mapping [45]. To identify key themes, all sticky notes were placed on a wall and two researchers (KS and DT) analyzed them thematically [46] by grouping them into clusters of similar themes and rearranging them as the analysis progressed. At the end, each cluster was described as a specific theme.

Notes from the prototype testing sessions (Study 2) and role plays (Study 4) were summarized. To identify wider themes relating to delivering therapy via technology and an integrated approach, we copied session notes onto a virtual board with interactive sticky notes [47], where three researchers (KS, DC, and CP) used affinity mapping [45] to create clusters of notes with similar topics and to identify key themes.

The interviews (Study 3) were transcribed, and the transcripts were analyzed inductively using a thematic approach [46]. First, three researchers (KS, DC, and CP) read the same two transcripts, discussed potential codes, and agreed to conduct open coding without a predefined coding frame. The transcripts were then uploaded to NVivo 11 (QSR International) for Mac, and one researcher (KS) coded the entire data set using a bottom-up approach, which involved coding the transcripts at the sentence level with detailed descriptive codes; no predefined coding guide was used. Codes and coded extracts were regularly reviewed throughout the coding stage. After all transcripts had been coded, the researchers discussed the codes and started collating them into themes using the virtual board with interactive post-it notes [47]. This led to the establishment of an initial set of themes, which were later reviewed together with the coded extracts, which led to further changes and discussions.

While all studies were analyzed separately, in the following sections, we report the combined results representing patients’ (Studies 1 and 2) and therapists’ (Studies 3 and 4) views and experiences.

## Results

### Studies 1 and 2: Findings From Design Workshops and Prototype Testing Sessions With People Who Received CBT for Depression in the Past

We identified three overlapping themes describing the key types of features for an integrated platform for delivering CBT for depression as follows: features that help to overcome depression-related barriers; features that support engagement; and features that reinforce learning and developing new skills. They are described below with illustrative quotes.

#### Patient Theme 1: Overcoming Depression-Related Barriers

Both studies highlighted participants’ awareness of internal barriers inherent to people living with depression and ways of overcoming them. As depression can make it difficult for people to engage with treatment, the technology for delivering CBT for depression should provide content that is simple and easy to find and understand, to reduce these barriers.

For me, I would like it to be simple, not too many things going on […] Non-busy, non-frightening, taking it in chunks, so you can absorb the information fairly easily.

Patient #14, 46-year-old female; prototype testing

Drawing from their past experiences, participants highlighted the danger of becoming overwhelmed and thus the need to manage expectations, to release materials as and when they become relevant and needed.

When we’re depressed, we don’t always feel like exploring.

Patient #15, 59-year-old female; prototype testing

Participants emphasized the importance of positive framing. With CBT’s focus on analyzing and reframing negative automatic thoughts, being able to, for example, record positive events, emphasize their goals, and see what they have achieved, was seen as important to motivate users and help them cope.

You’ve got there [a field to complete that asks] ‘what you want to get out of therapy’ which I think is a more positive than asking ‘what are you struggling with’ […] It’s much more positive to have it that way and I think every time you’ve gone to your profile page, you don’t want to be reminded of the things you’re struggling with.

Patient #16, 39-year-old male; prototype testing

#### Patient Theme 2: Supporting User Engagement

Workshop participants thought that the most important part of CBT was the relationship with the therapist; therefore, the features supporting mutual understanding and trust, as well as enabling the collaborative aspects of therapy (eg, completing worksheets together) were key to engaging people.

I would like some reassurance that I’m not just doing this and it’s been read. You should probably get some feedback and know that somebody will be listening, [that] it’s not just going to cyberspace.

Patient #1, 63-year-old female; workshop

Participants from both studies thought that enabling personalization and customization were important in supporting engagement, as they helped to identify with the treatment and made it more relevant. Allowing users to add their own notes and providing materials relevant to their situation were all mentioned as important factors in keeping people engaged with CBT.

I want MY personal page so that you know you’re not on just some general CBT page, you’re on your personal page that shows what I’ve already done and where I am.

Patient #6, 52-year-old female; workshop

Participants also emphasized the importance of features that help people stay on top of their treatment, for example, reminders to complete a homework task or prepare for the next therapy session. They also wanted to be able to track progress and be able to see how far they have come since the beginning of therapy.

I like the idea that I can track where you are and how much you have done. It’s not really how much you have to do, it’s about validating that you’ve done [it]

Patient #3, 59-year-old female; workshop

Feedback was mentioned as key in supporting engagement. Participants felt that it should be two-fold as follows: the therapist should be able to provide personalized feedback, and on another level, an automatic built-in feedback mechanism that confirms task completion would also be helpful, as users would know that their actions have been saved and acknowledged.

You could give people an incentive, a […] reward for just having done the thing. And then we wanted to distinguish that from getting feedback from therapist on what you’ve done, which is a bit different that just having done it. Automated rewards and proper feedback. People need both of those things in order to stay motivated.

Patient #3, 59-year-old female; workshop

#### Patient Theme 3: Supporting Learning and Acquisition of New Skills

Participants argued that to be effective, the technology should support the acquisition and maintenance of new skills. They believed that access to resources (eg, relevant reading materials or videos) would be crucial in supporting therapy. It would also allow people to revisit topics covered in earlier therapy sessions or after the therapy has ended.

I think CBT is not a one-off thing, I think it’s something you can use on and on and on, and if you’ve got this […] you can go back and see and revisit things, because life goes round and round in circles, really, and I think that’s something quite useful.

Patient #14, 46-year-old female; prototype testing

Participants believed that contact with the therapist was key in learning these skills, although there was no consensus with regard to the best way to contact the therapist. While some participants would prefer to receive therapy face-to-face, others liked the flexibility the online setting offered. The majority, however, agreed that meeting the therapist at least at the beginning is important to establish rapport and build the relationship.

I think you need to meet people and when you’re feeling vulnerable you need to understand how people convey themselves over email and over telephone. When you’re very emotional, you always take the worst-case scenario, so I think once you’ve met somebody and built that relationship… Maybe have the first [session] together and maybe one more later.

Patient #14, 46-year-old female; prototype testing

### Studies 3 and 4: Findings From Qualitative Interviews and Platform Role-Play Sessions With Therapists

The interviews with therapists provided broad themes and helped to understand the impact technology could have on their current practice. The role-play sessions enabled unpacking of the initial findings, as therapists were able to focus on delivering therapy via a new technology. We identified three key themes that are described below with illustrative quotes.

#### Therapist Theme 1: The Importance of the Therapist and Client Working Together

All therapists acknowledged the importance of face-to-face contact. They all believed that to build rapport, it is necessary to see the other person. As a result, they worried that online contact would limit additional cues and body language they relied on during therapy.

That feeling that you have in the room with someone I think is more powerful in a room than it is on the phone. I'm not saying that you don't get some of that on the phone, but I think that the information that you get is probably slightly different.

Therapist #8, 45-year-old female; interview

Despite being open to trying other types of contact and seeing the benefits of using technology to work and communicate with their clients, therapists worried that there would be less time to focus on the content of therapy as they would need to keep checking whether the client is engaging, looking at the right page, etc.

Doing that little role-play [...] it felt like there were many more things to be thinking about. It felt much more clunky than if I was sat face-to-face with a patient. It didn’t feel very natural and it didn’t feel particularly therapeutic. Just that, for instance, me saying to [T5] “Would you mind refreshing that page so I can see it?”, having to say to her “OK, I’m going to bring up on your screen a worksheet” [...] it didn’t feel as if it flowed in the same way as speaking and writing in a session.

Therapist #6, 30-year-old female; role-play session

Therapists had concerns about technology giving clients too much control and were worried they would want to focus on topics not related to therapy goals. While this can already happen in face-to-face therapy, technology could make it easier. At the same time, they thought that the online format could also make it easier for patients to take ownership of their treatment and facilitate engagement, which is necessary for positive outcomes.

I guess, yeah, the struggles in the past of computerized CBT has been this idea that it’s very… kind of having to fit the client into the program that’s already there and fit them into the boxes, whereas [integrated approach] sounds much more guided by the patient, there’s lots of elements to it. It’s not just kind of one strand, […] but much more idiosyncratic and lots more flexibility.

Therapist #6, 30-year-old female; interview

#### Therapist Theme 2: Impact of Technology on Therapists’ Workflow and Workload

All therapists agreed that introducing a new technology to their practice would change how they deliver therapy and would have an impact on their workload. In particular, they worried about extra work that they may need to do between therapy sessions.

If you’ve got sessions booked in you’ve got specific time slots, but I suppose therapists would have to think about how they allocate time to review worksheets and I just think that’s the kind of thing that could potentially add up.

Therapist #4, 36-year-old female; interview

In addition, they expressed concerns that if clients were able to message them between therapy sessions, it would not only have a negative impact on therapists’ workload but also complicate how they manage risk.

I think it's really good but [my concern] is whether people then start bombarding you with questions. Or if people are sending you stuff that is potentially like risk stuff. Someone was to send you a message to say “I'm feeling really suicidal” and that's not necessarily something you're going to pick up straight away

Therapist #9, 35-year-old female; interview

If the email is emotion-laden and talking about all sorts of problems […] I can imagine that feeling quite hard to deal with, and perhaps one getting a little bit worried that one isn’t providing a good service […] It feels like something [that] could be a little bit damaging to the therapist’s sense of well-being really, depending on how many come and what they’re like.

Therapist #7, 53-year-old male; interview

The use of technology could also lead to a positive change. Therapists reported that in their current practice, they often did not have enough time to prepare for sessions in advance. An integrated approach would make it easy to see whether clients have done their homework and what they would like to talk about, and to share their background details.

The more information the better, really, from the therapist’s point of view. […] So you would want to know of trauma and previous struggles with low mood and anxiety, and perhaps whether they had any previous therapy or CBT

Therapist #12, 34-year-old female; role-play session

Therapists also appreciated the ability to access digital resources by either party at any time. Having this shared space would allow them to easily locate and share materials, and track client engagement with these materials. Delivering therapy online also meant that session transcripts could potentially become therapeutic materials that clients could revisit at any time.

Having that kind of transcript would be really useful for the patient to be able to access in between sessions because it’s obviously serving as a useful prompt regarding what’s been discussed.

Therapist #6, 30-year-old female; interview

#### Therapist Theme 3: Supporting Clients’ Engagement With Therapy

Therapists reported that being able to see the same worksheet and doing things together would help clients understand complex topics and better engage with therapy. Asking clients to complete the worksheets themselves would increase accountability, although therapists would like to be able to step in and support clients if necessary.

It is good to encourage people to write things down themselves because this means that they’ve got that kind of control of what they do over there, quite active in a sense. But I wonder when people are very depressed, whether that’s quite a lot of effort and you can just as easily repeat something back to them and say ‘do you want this written down?’ and then do it for them.

Therapist #3, 32-year-old female; role-play session

Therapists also believed that technology could support clients’ motivation. This could be achieved by simplifying all tasks, providing reminders, making everything easily available, and reducing any frictions or barriers related to homework completion.

I'm just thinking about homework and how that comes into it, whether there's anything in between sessions to remind them to do it, what they're doing, or whether before a therapy session they need to just think about what they've done.

Therapist #9, 35-year-old female; interview

Finally, therapists considered the ability to see if the clients are logging in and doing their work as another tool for supporting engagement. However, they did acknowledge potential issues with this type of monitoring.

This might feel a bit like Big Brother if you mention that “you haven’t logged on for a week” […] But I mean, this is to be expected probably, isn’t it, it’s an online thing […] this could encourage them to engage more

Therapist #12, 34-year-old female; role-play session

## Discussion

### Principal Findings

The perspectives offered by people who received CBT in the past and therapists who deliver it provided valuable insights on the potential use of integrated therapy systems. We discuss these insights below and provide 12 generalizable design recommendations to support designers of integrated systems. These recommendations are summarized in Table 1.

**Table 1:** List of design considerations for blended systems that aim to further integrate online resources and contact with a therapist.

Area	Design considerations
Therapeutic relationship	1. Use face-to-face sessions to build rapport and trust, and online sessions to support skills development. 2. Allow therapists and patients to collaboratively work together on skill-related exercises, such as worksheets. 3. Keep a record of therapist-patient communication and make resources and transcripts available to patients well beyond the end of therapy, and/or enable the download of all materials for later use.
Personalized treatment	4. Provide a wide selection of exercises and worksheets and enable the therapist to select appropriate resources to offer to the patient. 5. Consider ways in which the therapist (and perhaps even the patient) could take ownership of such materials; potentially modifying and creating new worksheets in response to their particular preferences and needs.
Supporting learning	6. Support both therapist-directed and patient-led usage, within and between sessions: When therapist-directed, it should be clear to patients what is expected of them. Materials for immediate use within a session and to be used between sessions (eg, this week’s worksheet) should be in the foreground, together with expected tasks. At the same time, patients should have the option to explore materials that are relevant but not of immediate use.
Engagement and accountability	7. Make patient commitments explicit and allow them and their therapists to review and update progress on these commitments. 8. Use automated feedback as a positive reward for engagement, but do not use it as a substitute for personal feedback from the therapist. 9. Allow therapists to review and provide feedback on worksheets completed by patients between therapy sessions.
Changing context	10. Take into consideration the context in which therapists operate, as well as their workload, work patterns, and expectations. 11. Support between-session contact in a way that allows therapists to set and maintain boundaries and manage patients’ expectations. 12. Support risk management, but do not place responsibility on the therapists between therapy sessions. Instead, make it clear to patients where they can get help if they are distressed and require immediate support.

#### Support for the Therapeutic Relationship Versus Skills Coaching

Both patients and therapists reflected on the potential changing nature of the therapeutic relationship in the context of an integrated system. The major challenge they saw was the difficulty in creating a human and supportive environment remotely. Therapists were concerned that this would interfere with their ability to “read” the other person’s needs, whereas patients emphasized the importance of building rapport and trust early, which may not always be possible with online contact. These concerns are consistent with findings of a Delphi study and interviews by van der Vaart et al who explored the combination of face-to-face and online therapy [25]. They reported that some participants were concerned that limited face-to-face contact could weaken the patient-therapist bonding and lack of non-verbal communication could cause interpretation issues or lead to poor communication. However, there was an agreement that while the initial session would benefit most from face-to-face contact, others could be online. Moreover, there is evidence showing that using instant messaging to deliver and receive therapy is acceptable and can be effective [14-17].

At the same time, both groups recognized that an online system could actually make it easier to focus on the skill-based aspects of the work together and avoid the potential “trap” (from a CBT perspective) of “talking round and round stuff.” This is in line with findings in the study by van der Vaart et al [25] who noted that the online format is the best for the most practical aspects of therapy. In addition, existing research suggests that digital worksheets can support engagement with homework [12,13], but our results show that this could go further. Easy access to resources, creation of archives of past sessions, and keeping track of progress could all support long-term learning beyond the end of therapy. This leads to the following design considerations for systems that aim to further integrate online resources and contact with a therapist:

1. Use face-to-face sessions to build rapport and trust, and online sessions to support skills development.

2. Allow therapists and patients to collaboratively work together on skill-related exercises, such as worksheets.

3. Keep a record of therapist-patient communication and make resources and transcripts available to patients well beyond the end of therapy, and/or enable the download of all materials for later use.

#### The Value of Personalization and Flexibility

Both patients and therapists spoke of the need for flexibility in the choice of skills one could learn and materials one could access. In particular, it was important for patients to learn not only the skills that would help them with their depression in the short-term, but also how to use these new skills in the future. Participants emphasized the need for different formats of materials (video and text) to match different people’s needs. Participants also saw the expertise and intuition of the therapist in responding to patients’ needs as valuable, enabling them to tailor the treatment for each patient, which would make it more useful and more engaging. This was identified as a potentially relevant advantage of an integrated approach over versions of cCBT that tend to be inflexible [7]. This suggests that the acceptability of such a system (to both therapists and patients) would be strongly influenced by its ability to support this flexibility. Research into blended therapy and the evaluations of existing systems that include therapist involvement show that this may indeed be the case [22-25]. This finding is also consistent with other recent research on digital mental health outside of the CBT space, which again found that therapist-led [48] and patient-led tailoring [49] can help to increase patient engagement. Therefore, this leads to the following design considerations:

4. Provide a wide selection of exercises and worksheets and enable the therapist to select appropriate resources to offer to the patient.

5. Consider ways in which the therapist (and perhaps even the patient) could take ownership of such materials; potentially modifying and creating new worksheets in response to their particular preferences and needs.

#### Therapist-Led Versus Patient-Led Tailoring

Both therapists and patients commented on the personal nature of a therapeutic journey. Therapists emphasized the value of it being guided by the patient, while patients expressed concerns regarding the impact of severe depression on their motivation to do this. While this initially may appear contradictory, on closer observation, it is more complementary. Patients recognize that as they develop skills and become more confident, the responsibility moves toward them. This is a classic example of learning, with a period of support (*scaffolding*) by the expert leading to a growing confidence in the learner, allowing them to become independent [50]. This leads to the following design considerations:

6. Support both therapist-directed and patient-led usage, within and between sessions:

- When therapist-directed, it should be clear to patients what is expected of them. Materials for immediate use within a session and to be used between sessions (eg, this week’s worksheet) should be in the foreground, together with expected tasks.
- At the same time, patients should have the option to explore materials that are relevant but not of immediate use.

#### Engagement and Accountability

Both therapists and patients noted the potential benefits of therapists being able to see patient activity between sessions to encourage engagement, although they did have concerns about potential “big brother” aspects. Participants identified two different aspects of this monitoring relationship and noted that both have value. The first is a simple acknowledgment (possibly automated) that a patient has followed through on a commitment (such as filling in a worksheet), and the second is the human touch of the therapist actually looking at and reviewing the work. Combining automated and human feedback in this way has potential to be more engaging and leads to the following design considerations:

7. Make patient commitments explicit and allow them and their therapists to review and update progress on these commitments.

8. Use automated feedback as a positive reward for engagement, but do not use it as a substitute for personal feedback from the therapist.

9. Allow therapists to review and provide feedback on worksheets completed by patients between therapy sessions.

#### The Changing Role of the Therapist

Therapists recognized the change in their role and expectations that a more integrated system might bring and expressed concerns regarding this change. An integrated approach offers the possibility of a greater diversity of interactions within the therapeutic relationship. Some patients felt that simply having the online system always available would make their therapy more salient in their daily life, instead of being just a once-a-week contact. However, this obviously bears risks, which the therapists identified.

The first risk is related to workload. It is well known that technology can increase administrative burden without provision of extra time to carry it out [51]. An integrated system has the potential to create new work that is actually contributing to the therapeutic relationship, for example, responding to questions, reviewing worksheets a patient has completed, and sending an encouraging email to a patient who is not engaging. Therapists rightly highlighted the need to identify such work and timetable it explicitly into their day and workload, as demand for treatment is high [4,52]. The second risk is related to the nature of this between-session contact, as it can blur therapeutic boundaries, create an expectation for the patient that the therapist is always available, and make therapists feel more responsible for vulnerable patients. Any changes in the relationship induced by an integrated system must avoid an expectation on the patient’s part that it is the therapist’s job to manage risk between therapy sessions. This leads to the following design considerations:

10. Take into consideration the context in which therapists operate, as well as their workload, work patterns, and expectations.

11. Support between-session contact in a way that allows therapists to set and maintain boundaries and manage patients’ expectations.

12. Support risk management, but do not place responsibility on the therapists between therapy sessions. Instead, make it clear to patients where they can get help if they are distressed and require immediate support.

### Limitations and Future Work

As our research was qualitative in nature, we have engaged a relatively small number of participants. However, our participant number is consistent with user-centered design studies, and repeated evidence has shown that these methods can provide generalizable design guidelines [28,53-55]. As such, the research we conducted enabled us to gather design requirements and collect feedback on the prototype, which then informed the development of an integrated platform. The longitudinal evaluation study of the near-final version of the platform has been published previously [35].

 The majority of our participants were women. This may be because women are more likely than men to seek mental health treatment [56] and the IAPT workforce is predominantly female [52]. In terms of implications, it could mean that the resulting platform will better meet the treatment needs of women than men. However, male participants did take part in both the workshops and later prototype testing studies, and their opinions have also informed the design of the platform. In addition, none of the participants were from black, Asian, and minority ethnic (BAME) communities, and therefore, some of the findings might not reflect the views of these populations. We acknowledge that there are inequalities in access to mental health care that disproportionately affect BAME communities [56], leading to difficulties in recruiting members of BAME communities for research studies [57].

### Conclusions

By engaging end users and drawing from the user-centered design methods for eliciting design requirements, we identified 12 design considerations for developing integrated therapy systems. To meet users’ needs, such systems should be able to help retain the personal connection between the therapist and the client; support both therapist- and patient-led activities; and provide access to materials and should ensure the ability to monitor progress. However, developers of such systems should be mindful of their capacity to disrupt current work practices and increase therapists’ workload. Future work should evaluate the clinical effectiveness and cost-effectiveness of integrated systems in a real-world context, including barriers and enablers of implementing such systems, as well as the impact of different design decisions on delivering treatment in primary care settings.

## Appendix

Multimedia Appendix 1 Information about the participants who attended design workshops and prototype testing sessions.

Multimedia Appendix 2 Information about the therapists who attended role plays and interviews.

Multimedia Appendix 3 Study materials used during design workshops (Study 1).

Multimedia Appendix 4 Study materials used during prototype testing (Study 2).

Multimedia Appendix 5 Study materials used during interviews (Study 3).

Multimedia Appendix 6 Study materials used during role plays (Study 4).

## Abbreviations

BAME: black, Asian, and minority ethnic
CBT: cognitive behavioral therapy
cCBT: computerized cognitive behavioral therapy
IAPT: Improving Access to Psychological Therapies
NHS: National Health Service
NICE: National Institute for Health and Clinical Excellence
RCT: randomized controlled trial
