# Supplementary material for: Design Considerations for the Integrated Delivery of Cognitive Behavioral Therapy for Depression: User-Centered Design Study
Source: JMIR Ment Health. 2020 Sep 3;7(9):e15972. doi: 10.2196/15972 (PMC7499168; doi:10.2196/15972)
Supplement: Multimedia Appendix 1 [file mental_v7i9e15972_app1.pdf]

Appendix 1. Information about the participants who attended design workshops and prototype testing sessions

**Characteristics**	**All participants (N=18)**	**Those who attended workshops (N=12)**	**Those who attended prototype testing sessions (N=7)**
Age (years)			
Mean	48.5	48.2	50.9
SD	13.4	14.3	11.4
Gender: n (%)			
Female	13 (72%)	9 (75%)	4 (57%)
Male	5 (28%)	3 (25%)	3 (43%)
Ethnicity: n(%)			
White	18 (100%)	12 (100%)	7 (100%)
Marital status: n(%)			
Married/Living as married	5 (28%)	4 (33%)	1 (14%)
Single	6 (33%)	3 (25%)	3 (43%)
Separated/Divorced	8 (44%)	6 (50%)	3 (43%)
Education: n(%)			
Degree or equivalent	8 (44%)	5 (42%)	3 (43%)
HNC, HND, SVQ (Level 4 or 5) or RSA Higher Diploma	2 (11%)	4 (33%)	1 (14%)
A-level, Higher Grade, or equivalent (GNVQ/NVQ Advanced, GSVQ/SVQ (Level 3) or RSA Advanced Diploma)	2 (11%)	1 (8%)	1 (14%)
GCSE, Standard Grade, O-level or equivalent (GNVQ/NVQ Intermediate or Foundation, GSVQ (Level 1 or 2) or RSA Diploma)	6 (33%)	4 (33%)	2 (29%)
No formal qualifications	-	-	-
Age first aware of problems with depression: n (%)			
Less than 18 years old	3 (17%)	3 (25%)	-
18-29 years old	5 (28%)	4 (33%)	1 (14%)
30-39 years old	6 (33%)	3 (25%)	4 (57%)
40-49 years old	2 (11%)	1 (8%)	1 (14%)
50-59 years old	2 (11%)	1 (8%)	1 (14%)
60-69 years old	-	-	-
70+ years old	-	-	-

Number of separate episodes of depression experienced			
1 time	1 (6%)	1 (8%)	-
2-4 times	3 (17%)	3 (25%)	-
5+ times	14 (78%)	8 (67%)	7 (100%)
Mental health over the last 2 weeks			
Not depressed	6 (33%)	5 (42%)	1 (14%)
Slightly depressed	6 (33%)	2 (17%)	4 (57%)
Moderately depressed	6 (33%)	5 (42%)	2 (29%)
Severely depressed	-	-	-
Extremely depressed	-	-	-
Impact of current depression on usual activities:[1] *n (%)*			
No problem doing usual activities	2 (11%)	2 (17%)	-
Slight problems doing usual activities	8 (44%)	5 (42%)	3 (43%)
Moderate problems doing usual activities	3 (17%)	2 (17%)	1 (14%)
Severe problems doing usual activities	3 (17%)	2 (17%)	2 (29%)
Unable to do usual activities	-	-	-
On antidepressant medication:[2] *n (%)*			
Yes	10 (56%)	7 (58%)	3 (43%)
No	8 (44%)	5 (42%)	4 (57%)
Ever used computerized CBT: n (%)			
Yes	4 (22%)	4 (33%)	1 (14%)
No	14 (78%)	8 (67%)	6 (86%)
Participant recruited from: n (%)			
Previous trial	8 (44%)	7 (58%)	1 (14%)
Local services	10 (56%)	5 (42%)	6 (86%)

Footnotes:

1. Two participants didn't answer this question
2. One participant reported that GP prescribed them HRT medications for their depression, but they were not actually antidepressants
