# Supplementary material for: Design Considerations for the Integrated Delivery of Cognitive Behavioral Therapy for Depression: User-Centered Design Study
Source: JMIR Ment Health. 2020 Sep 3;7(9):e15972. doi: 10.2196/15972 (PMC7499168; doi:10.2196/15972)
Supplement: Multimedia Appendix 2 [file mental_v7i9e15972_app2.pdf]

Appendix 2. Information about the therapists who attended role-plays and interviews

**Characteristics**	**All therapists (N=12)**	**Those who attended role-plays (N=5)**	**Those who attended interviews (N=11)**
Age (in years)			
Mean	42.8	39.6	43.5
SD	8.8	10.1	8.8
Gender: n (%)			
Female	9 (75%)	5 (100%)	8 (73%)
Male	3 (25%)	-	3 (27%)
Experience as CBT therapist (in years)			
Mean age	8.1	5.2	8.2
SD	5.0	1.8	5.2
Professional background:[1] *n (%)*			
CBT therapist	8 (67%)	4 (80%)	7 (64%)
Clinical psychologist	3 (25%)	1 (20%)	3 (27%)
Occupational therapist	1 (8%)	-	1 (9%)
Counsellor/Counselling psychologist	1 (8%)	-	1 (9%)
Nurse	2 (17%)	-	2 (18%)
Social worker	1 (8%)	1 (20%)	1 (9%)
Accredited by British Association for Behavioural and Cognitive Psychotherapies: n (%)			
Yes	10 (83%)	4 (80%)	9 (82%)
No	2 (17%)	1 (20%)	2 (18%)
Works within IAPT services: n (%)			
Yes	11 (92%)	5 (100%)	10 (91%)
No	1 (8%)	-	1 (9%)
Experience as a supervisor: n (%)			
Yes	11 (92%)	4 (80%)	10 (91%)
No	1 (8%)	1 (20%)	1 (9%)
Work sector: n (%)			
NHS	10 (83%)	5 (100%)	9 (82%)
Private sector	1 (8%)	-	1 (9%)
Both	1 (8%)	-	1 (9%)

Experience with delivering computerized CBT:[2] n (%)			
None	4 (33%)	3 (60%)	3 (27%)
Beating the Blues	4 (33%)	2 (40%)	4 (36%)
COPE	-	-	-
MoodGym	1 (8%)	1 (20%)	1 (9%)
Living to the Full	4 (33%)	1 (20%)	4 (36%)
IESO	-	-	-
SilverCloud	2 (17%)	1 (20%)	2 (18%)
Other	3 (25%)	1 (20%)	3 (27%)

Footnotes:

1. Four participants selected more than one answer
2. Other includes Fear Fighter, Centre for Clinical Interventions, “Youtube”, “Jim White”
