# Supplementary material for: Design Considerations for the Integrated Delivery of Cognitive Behavioral Therapy for Depression: User-Centered Design Study
Source: JMIR Ment Health. 2020 Sep 3;7(9):e15972. doi: 10.2196/15972 (PMC7499168; doi:10.2196/15972)
Supplement: Multimedia Appendix 3 [file mental_v7i9e15972_app3.pdf]

Appendix 3. Study materials used during design workshops (Study 1)

Access workshops

Activity guide:

1. Warm up sketching exercise
2. Barriers to access (to CBT in general and cCBT in particular)
    a. Groups prepare lists
    b. Groups discuss the barriers
3. Overcoming the barriers
    a. Groups prepare lists of ideas
    b. General discussion about overcoming the barriers, including identifying barriers that could be addressed by technology
4. Designing an online platform for CBT
    a. Groups discuss key features a system would need to support CBT
    b. Groups sketch their ideas
    c. Groups present their sketches
    d. General discussion about online CBT, features, etc.

Engagement workshops

Patient personas:

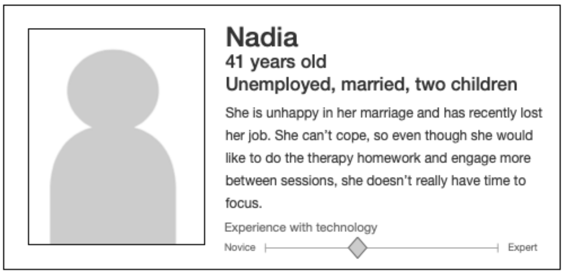

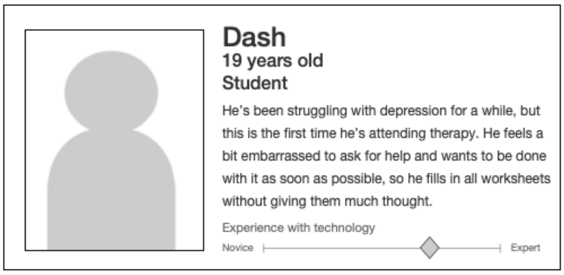

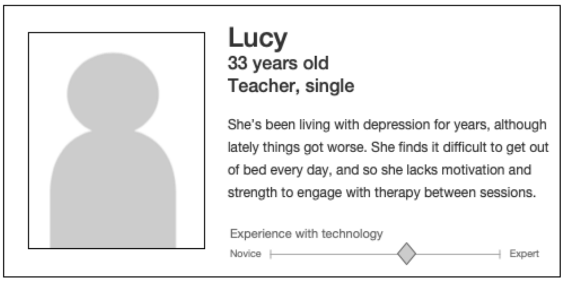

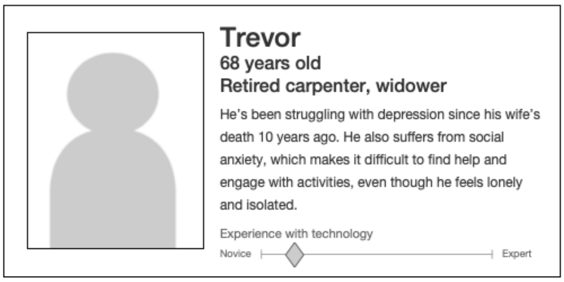

NB. The personas used during workshops had pictures representing real people. However, while the photo license allowed use to them during research, we were

unable to use them as part of this publication and had to replace them with placeholders.

Activity guide:

1. Warm up sketching exercise
2. Barriers to engagement (with CBT and with cCBT)
    a. Groups prepare lists
    b. Groups discuss the barriers
3. Overcoming the barriers
    a. Groups prepare lists of ideas
    b. General discussion about overcoming the barriers and supporting engagement, including identifying barriers that could be addressed by technology and how technology could help people stay engaged with their therapy
4. Designing an engaging platform for CBT
    a. Groups discuss key features a system would need to have to help people engage with CBT
    b. Groups sketch their ideas
    c. Groups present their sketches
    d. General discussion about online CBT, engagement, factors that would make people disengage, etc.
