# Supplementary material for: Design Considerations for the Integrated Delivery of Cognitive Behavioral Therapy for Depression: User-Centered Design Study
Source: JMIR Ment Health. 2020 Sep 3;7(9):e15972. doi: 10.2196/15972 (PMC7499168; doi:10.2196/15972)
Supplement: Multimedia Appendix 4 [file mental_v7i9e15972_app4.pdf]

Appendix 4. Study materials used during prototype testing (Study 2)

Round 1 – session script

Introduction
During this session, I will ask you to complete specific tasks that will help us find what works and what needs to be improved with an online CBT platform. I will also show you some pages to get your feedback. Remember: I'm testing the platform, not you, so there are no right or wrong answers. If something doesn't make sense, that means we need to fix it, so let me know. I didn't build this website, so you won't hurt my feelings – you can be as critical as you want.

When doing the tasks, I would like you to talk aloud so that I know what you're thinking, what makes you hesitate, what bits you like. Some people find it a bit weird, so let's practice that first. I will show you a popular travel site and we will evaluate it, and after the practice we will move on to testing the platform, is that okay?

Practice task
This is Wikitravel (http://wikitravel.org/en/Main_Page). Can you tell me what do you think you can do here? What is this site for?

Now imagine you're planning to go to Porto for a week and want to find out more about the city, especially you would like to know how safe it is. Please tell me how you would go about finding this information.

First use

1. Let's pretend you have been referred to a new CBT treatment. This is the site where you need to log in before your first therapy session.
    a. What is your first impression?
    b. What do you expect to see there?
2. *[log in as Alicia for the participant]* Now look at this page, don't click anything just yet.
    a. Can you describe each section to me?
    b. What do you think you can do there? What is it for?

After the first therapy session
Imagine that you've just got home from the first session with your therapist. The first session was face-to-face, but the next one will be online using this platform. *[log in as Denarian for the participant]*

1. Have a look around the home page – there are a few new things here. Before we do anything, please tell:
    a. What do you expect to happen if you click on these new elements?

b. What do you expect to find in these new sections?

During your face-to-face session, you agreed with your therapist the date of your next session and the therapist promised to share some materials with you.

1. Find out the time of your next session.

The therapist also pointed out during the session that you tend to catastrophise a lot and said he would send you some materials to read.

a. Please describe how you would go about finding out this information.

Worksheets and worksheet sharing
Let's imagine you're a few weeks into your therapy *[log in as Arthur for the participant]*. You've been working on a few different worksheets, let's look at them in more detail.

1. Please open My Thought Review. Describe your first impressions. What do you think is going on here? Look around the page and describe what you see and think.

   a. Are the instructions clear?

   b. What do you expect to happen when you click each of the buttons?

   c. Look at the example – what do you think about it?

2. Let's now try to fill in a worksheet. Please open Simple Thought Record.

   a. Fill in the worksheet *[show Scenario 1]*.

   b. Share the first entry with therapist.

   c. What do you think will happen now that you've shared the worksheet with the therapist?

Mobile phone worksheet sharing
Let's test how the platform works on a phone. Let's imagine we're Arthur again. His therapist asked him to complete the Mood diary every day before the next session, especially when something makes him feel bad. *[show Scenario 2]*

1. Log in to the platform on the phone to start recording what happened.

2. Log in to the platform on the laptop to add details to your entry.

Feedback on storyboards

1. Compare both design styles – which one do you think is more appropriate?

SUS questionnaire
Thinking about all pages you have seen and the platform overall, please complete this questionnaire. Please read each statement aloud and explain your answer.

Round 2 – session script

Introduction
During this session, I will ask you to complete specific tasks that will help us find what works and what needs to be improved with an online CBT platform. I will also show you some pages to get your feedback. Remember: I'm testing the platform, not you, so there are no right or wrong answers. If something doesn't make sense, that means we need to fix it, so let me know. I didn't build this website, so you won't hurt my feelings – you can be as critical as you want.

When doing the tasks, I would like you to talk aloud so that I know what you're thinking, what makes you hesitate, what bits you like. Some people find it a bit weird, so let's practice that first. I will show you a popular travel site and we will evaluate it, and after the practice we will move on to testing the platform, is that okay?

Practice task
This is Wikitravel (http://wikitravel.org/en/Main_Page). Can you tell me what do you think you can do here? What is this site for?

Now imagine you're planning to go to Athens for a week and want to find out more about the city, especially you would like to know *how safe it is*. Please tell me how you would go about finding this information.

First use

1. Let's pretend you have been referred to a new CBT treatment. This is the site where you need to log in before your first therapy session.
    a. What is your first impression?
    b. What do you expect to see there?
2. *[log in as Alicia for the participant]* Now look at this page, don't click anything just yet.
    a. Can you describe each section to me?
    b. What do you think you can do there? What is it for?
3. Prepare for the first session:
    a. Have a look at the Library. Feel free to open various resources there. Can you talk us through what you see there?
    b. Complete your profile *[show user persona]*

After the first therapy session
Imagine that you've just got home from the first session with your therapist. The first session was face-to-face, but the next one will be online using this platform. *[log in as Denarian for the participant]*

1. Have a look around the home page – there are a few new things here. Before we do anything, please tell us:
    a. What do you expect to happen if you click on these new elements?
    b. What do you expect to find in these new sections?
    c. Can you look at the worksheets your therapist has shared with you? Please have a look at each of them and describe them to us.

During your face-to-face session, you agreed with your therapist the date of your next session and the therapist promised to share some materials with you. Let's imagine you're Denarian *[show user persona].*

1. Prepare for your next session:
    a. Complete the depression questionnaire
    b. Prepare session agenda
2. The therapist also mentioned during the session that you can find some reading materials on the platform and recommended you read a bit about sleep issues.
    a. Please describe how you would go about finding out this information.

Worksheets and worksheet sharing
Let's imagine you're a few weeks into your therapy [log in as Arthur for the participant] and you agreed to work on a worksheet at home.

1. Let's now try to fill in a worksheet. Please open Simple Thought Record.
    d. Fill in the worksheet *[show Scenario 1]*.
    e. Share the first entry with therapist.
    f. What do you think will happen now that you've shared the worksheet with the therapist?
    g. *[send feedback]* Find the comments your therapist has sent you.

Mobile phone worksheet sharing
Let's test how the platform works on a phone. Let's imagine we're Arthur again. His therapist asked him to complete the Mood diary every day before the next session, especially when something makes him feel bad. *[show Scenario 2]*

1. Log in to the platform on the phone to start recording what happened.
2. Log in to the platform on the laptop to add details to your entry.
3. Let's have a look at all the other worksheets on the phone.

SUS questionnaire
Thinking about all pages you have seen and the platform overall, please complete this questionnaire. Please read each statement aloud and explain your answer.

Round 3 – session script

Introduction
During this session, I will ask you to complete specific tasks that will help us find what works and what needs to be improved with an online CBT platform. I will also show you some pages to get your feedback. Remember: I'm testing the platform, not you, so there are no right or wrong answers. If something doesn't make sense, that means we need to fix it, so let me know. I didn't build this website, so you won't hurt my feelings – you can be as critical as you want.

When doing the tasks, I would like you to talk aloud so that I know what you're thinking, what makes you hesitate, what bits you like. Some people find it a bit weird, so let's practice that first. I will show you a popular travel site and we will evaluate it, and after the practice we will move on to testing the platform, is that okay?

Practice task
This is Wikitravel (http://wikitravel.org/en/Main_Page). Can you tell me what do you think you can do here? What is this site for?

Now imagine you're planning to go to Tokyo for a week and want to find out more about the city, especially you would like to know *how safe it is*. Please tell me how you would go about finding this information.

First use

1. Let's pretend you have been referred to a new CBT treatment. This is the site where you need to log in before your first therapy session.
    a. What is your first impression?
    b. What do you expect to see there?
2. *[log in as Alicia]* Now look at this page, don't click anything just yet.
    a. Can you describe each section to me?
    b. What do you think you can do there? What is it for?
    c. Can you prepare for the first session? *[show user persona]*
3. Let's look at preparations for the first session *[schedule a face-to-face session for tomorrow]*. You don't need to fill anything in, just look at each section and describe what you see and what you think.
    a. Describe the depression questionnaire
    b. Describe the session preparation questions

Online therapy session
Let’s have a look at how online sessions will work. They require less input than the first session.

1. Pretend you’re Mandy and prepare for your next session *[show patient profile]*:

    b. Complete the depression questionnaire

    c. Prepare session agenda

2. Let’s have a look at the online session screen.

    h. Please describe what you can see here.

    i. Let’s test the chat function. Pretend to be Mandy.

    j. Therapist has shared a worksheet with Mandy *[share Simple Thought Record]*. Can you fill it in for her?

    k. Let’s write session notes.

    l. Overall, what do you think about the online session?

Inbox and messages
Let’s have a look at the inbox.

4. Describe what you see and what you think about all elements.

5. Let’s pretend Mandy needs to contact her therapist to ask for clarifications regarding the worksheet.

    a. Describe how you would do it.

SUS questionnaire
Thinking about all pages you have seen and the platform overall, please complete this questionnaire. Please read each statement aloud and explain your answer.

Patient scenarios

Scenario 1
Arthur’s wife used to take care of the garden before she’s passed away, but since then the garden has been neglected. Arthur’s therapist suggested that Arthur may want to go sit outside in the sun instead of sitting at home in front of the telly all the time. Yesterday, he finally decided to slowly start cleaning things up. His neighbour noticed it and cheerfully shouted from behind the fence: “Finally! Looks like you’re moving on! This garden badly needs some work!”. Arthur didn’t like this comment, he felt angry, hurt, and embarrassed. He doesn’t want to move on and forget his wife! He doesn’t want to clean up his memories of her! He threw away a pot he was holding and stormed home. He didn’t leave the bed until the evening.

Task: Imagine you’re Arthur and need to record this event in the Simple Thought Record.

Scenario 2
Arthur is on a bus, sitting near the end and staring out of the window. At one stop, two women sit down in the seat before him and start gossiping loudly. They don't like it that their friend just sits at home and doesn't want to go anywhere, they would like to spend more time with her. Suddenly Arthur feels sad, because he also sits at home alone. "But that's fine", Arthur thinks, "no one would want to meet me anyway, no one cares, I'm useless." This makes him feel even worse.

Task: Imagine you're Arthur. You remember that you're supposed to be recording situations like that. Start recording this event to your Mood Diary on the phone, then switch back to the laptop and add more details.

Patient personas

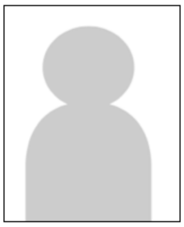

Alicia
41 years old
Unemployed, married, two children

She is unhappy in her marriage and has recently lost her job. Everything is overwhelming and she feels like she's not coping. She used to go swimming twice a week, but because of her depression she hasn't seen her friends form the local pool for nearly 4 months.

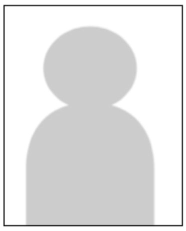

Denarian
19 years old
Student

He's been struggling with depression for a while, but this is the first time he's attending therapy. He feels a bit embarrassed to ask for help, but everything is overwhelming and he's not coping with studies. On top of that, he finds it difficult to sleep, which has negative impact on pretty much everything.

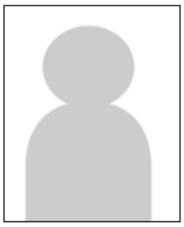

Mandy
33 years old
Sales executive, single

She's been living with depression for years, although lately things got worse because she's overwhelmed with work and doesn't get along with a new boss. She finds it difficult to get out of bed every day so has been calling in sick lately.

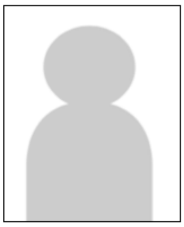

Arthur
68 years old
Retired carpenter, widower

He's been struggling with depression since his wife's death 10 years ago. He also suffers from social anxiety, which makes it difficult to find help and engage with activities, even though he feels lonely and isolated.

NB. The personas used during workshops had pictures representing real people. However, while the photo license allowed use to them during research, we were unable to use them as part of this publication and had to replace them with placeholders.

Patient profile

Background:
Mandy's manager joined their company a few weeks ago. During his first week, she was late a few meetings with him because she finds it difficult to get out of bed before depression. He wasn't happy about it, but she didn't mention her problems. Overall, she's struggling with depression but she doesn't want to talk about it to anyone at work.

What happened since the last session:
Last week she didn't finish writing a report on time and missed a deadline. Then, a couple of days later, her boss asked her to schedule a 1-on-1 meeting with him, but didn't say why. She called in sick the day after because she's worried he's going to fire her. Mandy hasn't been in the office since then and spent the last few days in bed.

Mandy's Therapy Aims
What do you want help with?
I need help with my depression. I can't focus on work and I can't get out of bed. This causes me stress at work.

What do you want to be different after therapy?
I want to be able to manage my work better and get up from bed without this being a terrible struggle. I don't want to lose my job

SUS questionnaire

Please think about the platform you've been using during this session and check the box that reflects your immediate response to each statement.

1. I think that I would like to use this platform frequently.

Strongly disagree	1	2	3	4	5	Strongly agree

2. I found it unnecessarily complex.

Strongly disagree	1	2	3	4	5	Strongly agree

3. I thought it was easy to use.

Strongly disagree	1	2	3	4	5	Strongly agree

4. I think that I would need the support of a technical person to be able to use it.

Strongly disagree	1	2	3	4	5	Strongly agree

5. I found the various functions in the platform were well integrated.

Strongly disagree	1	2	3	4	5	Strongly agree

6. I thought there was too much inconsistency.

Strongly disagree	1	2	3	4	5	Strongly agree

7. I imagine that most people would learn to use this platform very quickly.

Strongly disagree	1	2	3	4	5	Strongly agree

8. I found it very awkward to use.

Strongly disagree	1	2	3	4	5	Strongly agree

9. I felt very confident using the platform.

Strongly disagree	1	2	3	4	5	Strongly agree

10. I needed to learn a lot of things before I could get going with this platform.

Strongly disagree	1	2	3	4	5	Strongly agree

Prototype screenshots

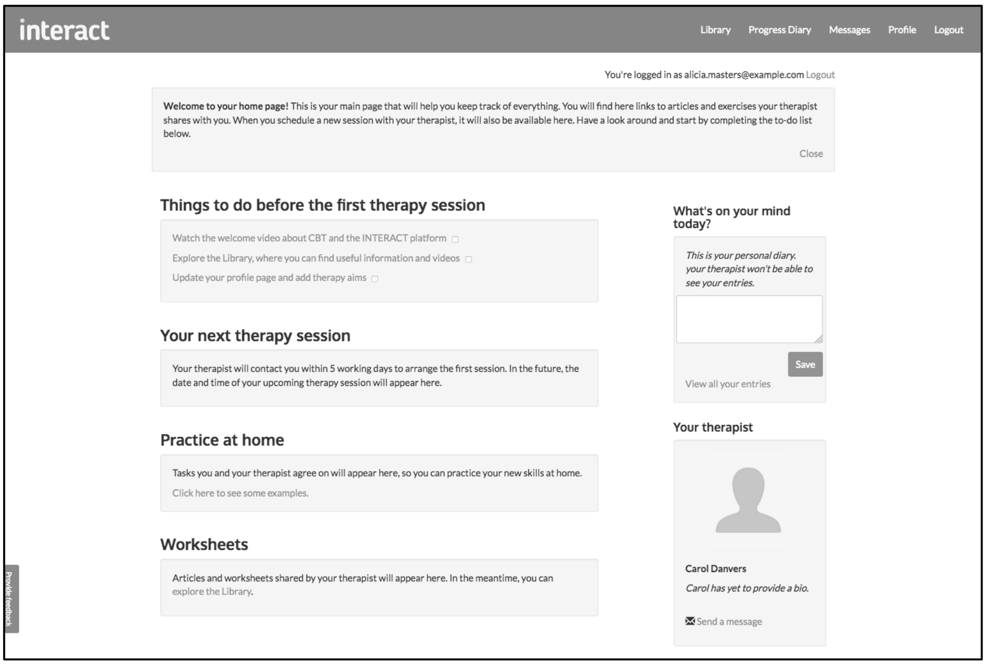

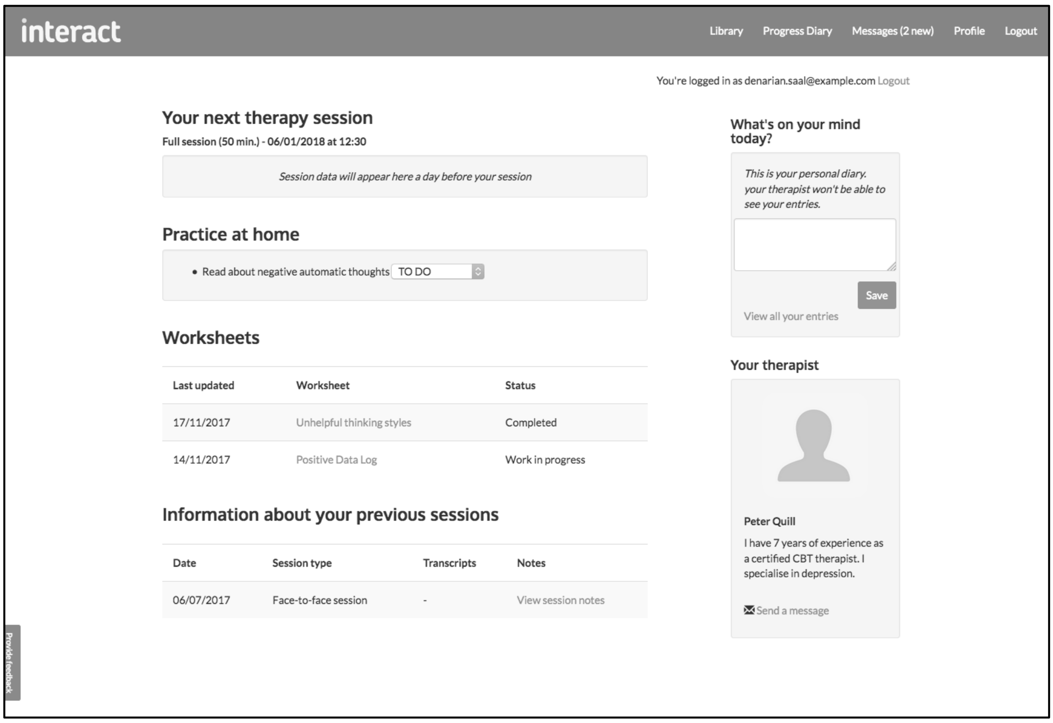

My Thought Review

Look at the Five Areas Assessment in the figure below. Write in all the things you have noticed in your life. These are possible targets for change.

AREA 1: Life situations, relationships and practical problems

AREA 2: Altered thinking

AREA 3: Altered feelings/emotions

e.g low, anxious, angry, ashamed, guilty

AREA 4: Altered physical symptoms/bodily sensations

AREA 5: Altered behaviour/activity levels

What do I do differently? Have I cut down/stopped any activities, or started doing something different

Your profile

First name *

Sylvie

Last name *

Lushton

Email address *

enchantress@example.com

Primary phone number (preferred) *

197289756122

Secondary phone number

Choose File no file selected

Why do you need a photo or picture? It will be used during online sessions. Click here to see how its going to look.

Therapy Aims

To help us help you, please try to answer the following questions before the first therapy session. You can edit or expand your answers at any time.

What do you want help with? *

What do you want to be different after therapy? *

Try to be specific. Click here to see example goals others have selected.

If there's anything in your background that you think your therapist should know, write it here

Save changes | Change Email Address | Change Password

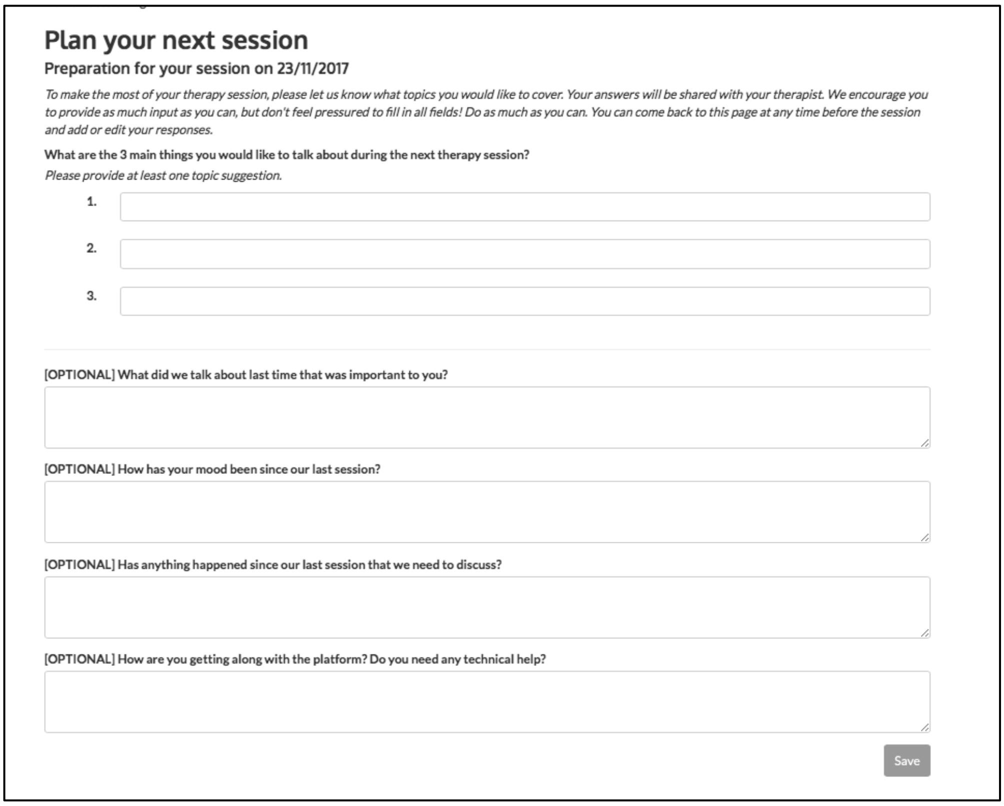

Plan your next session

Preparation for your session on 23/11/2017

To make the most of your therapy session, please let us know what topics you would like to cover. Your answers will be shared with your therapist. We encourage you to provide as much input as you can, but don't feel pressured to fill in all fields! Do as much as you can. You can come back to this page at any time before the session and add or edit your responses.

What are the 3 main things you would like to talk about during the next therapy session?

Please provide at least one topic suggestion.

1.

2.

3.

[OPTIONAL] What did we talk about last time that was important to you?

[OPTIONAL] How has your mood been since our last session?

[OPTIONAL] Has anything happened since our last session that we need to discuss?

[OPTIONAL] How are you getting along with the platform? Do you need any technical help?

Save

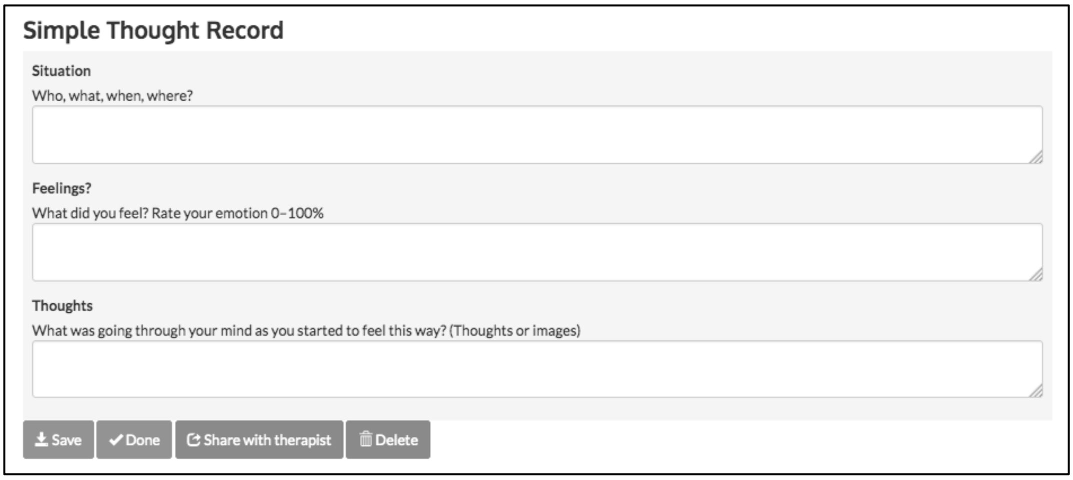

Simple Thought Record

Situation

Who, what, when, where?

Feelings?

What did you feel? Rate your emotion 0–100%

Thoughts

What was going through your mind as you started to feel this way? (Thoughts or images)

Save Done Share with therapist Delete
