# Supplementary material for: Design Considerations for the Integrated Delivery of Cognitive Behavioral Therapy for Depression: User-Centered Design Study
Source: JMIR Ment Health. 2020 Sep 3;7(9):e15972. doi: 10.2196/15972 (PMC7499168; doi:10.2196/15972)
Supplement: Multimedia Appendix 5 [file mental_v7i9e15972_app5.pdf]

Appendix 5. Study materials used during interviews (Study 3)

Interview guide

Part 1: Your experience

1. Can you tell me a bit about your background?
   (e.g. describe the current role, how long you've been working as a therapist, which patient groups you mainly work with, etc.)

2. Could you describe a typical day at work?
   (e.g. where you normally hold therapy sessions, how often you move between sites, whether you share an office, what are the main tasks, how much admin work, etc.)

3. What devices do you have access to at work? What devices do you use at work or for work related tasks? *(e.g. desktop PC, laptop, tablet, phone)*

4. Could you describe a typical therapy session?
   (e.g. use of worksheets, protocol to follow, differences in approach between patients)

5. Could you tell me a bit more about what you and the client do between the sessions?
   (e.g. use of worksheets – collaboration vs. homework, differences in approach between patients, engagement)

6. Do you ever hold therapy sessions remotely using instant messaging, telephone, Skype or other means? Why / why not? *(attitudes towards instant messaging vs. video vs. voice)*

7. How do you help clients stay engaged with the therapy?
   (e.g. contact between sessions, do you give patients materials to work on, do you suggest websites or apps to use)

8. Could you describe your clients' attitudes to homework?
   (e.g. whether they do it, what they find helpful, what the barriers are to them completing homework)

Part 2: Views on integrated therapy

Technology offers the opportunity to enhance the delivery of *high intensity CBT*. Our research programme is aiming to develop an integrated approach to therapy that combines direct contact with the therapist with online resources.

We know that clients who engage with homework have better outcomes. Therefore, the focus for us is on developing a platform that enables communication between the therapist and client, and encourages clients to do more independent work (homework) using worksheets (such as these available on websites like Get Self Help or Psychology Tools) and psychoeducational materials.

<Show and describe a diagram summarising our approach and illustrating therapy flow> Our plan is for the therapy to start with a face-to-face session and then subsequent therapy sessions would be held online using the platform we're

designing. There would be about 6 regular (hour long) therapy sessions and, later in therapy, a small number of (15-20 min.) "check-in" sessions between hour-long therapy sessions to facilitate engagement and briefly review progress with homework/between-session tasks, and also check for any problems using the platform. We expect that therapy sessions will take place weekly for the first four weeks but then may be spaced at longer intervals.

9. How does this new way of delivering CBT sound to you?
   (first impressions, concerns, advantages of this approach)

10. What are your views on the 'check-in' sessions?
   (e.g. their focus on facilitating engagement, reviewing progress, providing feedback on worksheets and/or homework)

<Turn the diagram page to show more details and describe relevant part before asking each question> Before an online therapy session, the client would be asked to log into the platform, complete a depression questionnaire (PHQ-9), set an agenda for their next session and share that information with their therapist.

11. What information would you like to see before the start of a session?

During the session, the therapist may introduce the client to a worksheet or psychoeducational material within the platform that links to the issues being discussed. The therapist and client would work through the worksheet together online in real-time so that the client becomes familiar with the worksheet and has a chance to practice filling in parts of the worksheet before they do this as part of their (between-session) homework. This communication between the client and therapist would primarily use instant messaging, but voice communication would also be available, especially when filling a worksheet together.

12. What do you think of the idea of working together with the client on worksheets online during the session?
   (e.g. fit into existing approach to therapy, IM vs. voice, devices used, access to microphone and/or headphones, concerns)

At the end of the session, the therapist and client would write a summary of the session together, and agree some things for the client to try out before the next therapy session.

13. What do you think about writing a session summary collaboratively with the client? *(e.g. usefulness for client and therapist, fit into existing approach to therapy, IM vs. voice, devices used, access to microphone and/or headphones*

Between sessions, patients would be expected to log into the system to complete their worksheets and/or read psychoeducational materials, and to share completed worksheets with the therapist. Therapists would be expected to review and comment on completed worksheets – either before or during a therapy session. Reviewing worksheets would be an important part of the short "check-in" sessions.

14. Therapist would need to review and comment on worksheets shared by the client and potentially respond to their queries. What do you think about that?
   (workload, potential issues, boundaries)

We would be able to record clients' interactions with the platform and shared materials, how often they log in, etc.

15. In terms of client monitoring, is there anything you would like to know about client's engagement with therapy?
(e.g. worksheet progress monitoring, notifications on client's interactions with the platform and shared materials)

16. Are there any tools you think would be useful in helping therapists to motivate and engage clients?
(e.g. reminders to patients, direct messages)

17. Overall, do you think that your clients would find this way of delivering CBT acceptable? Why / why not?

Part 3: Wrap-up

18. Do you have any other comments about using technology enhance the delivery of high intensity CBT for depression?
(e.g. features that could help to reduce your workload, potential issues)

19. To finish off, I would like you to open this diagnostic link <http://supportdetails.com> on all devices you use for work. It records only general information about the technology (e.g. browser type, operating system, size of your screen), no personal or identifiable information is recorded. It will help us understand what are the limitations of existing equipment used within the NHS.

Therapy flow overview

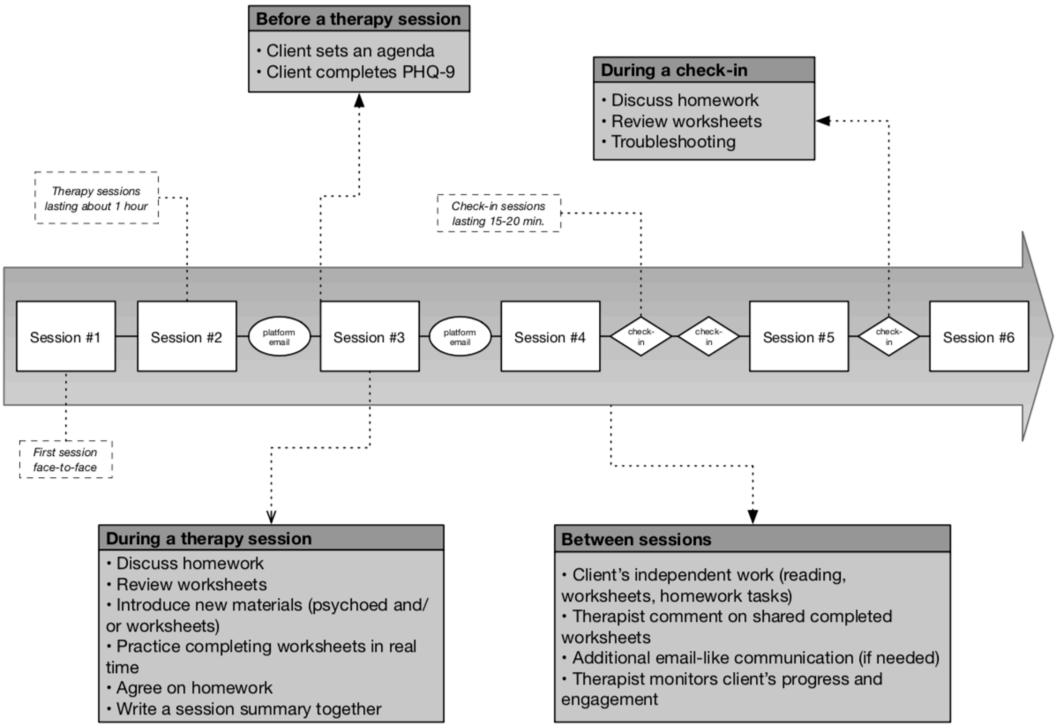

Example paper prototypes

HOME | LIBRARY | THERAPY SESSIONS | TRAINING MATERIALS | MESSAGES (2 new) | ACCOUNT SETTINGS | Log out

Hello, Joelle Smith

Your active clients

	Client	Latest PHQ-9 score (change)	Next session	Last login	Worksheets to review	Messages
!	Kamala Khan	23 (+2)	Arrange session	02/06/2017	none	none
!	Elsa Bloodstone	17 (+4)	27/07/2017 at 17:00	23/06/2017	1 worksheet	none
	Tony Stark	18 (+5)	21/07/2017 at 13:30	30/05/2017	none	none
	Sansa Stark	20 (+4)	Arrange session	01/07/2017	none	none
	**Joanna Blablason**	**15 (-)**	**Today at 15:00**	**02/06/2017**	**4 worksheets**	none
	Elliott Spider	17 (-2)	19/07/2017 at 15:45	23/05/2017	none	none
	Squirrel Queen	8 (+3)	20/07/2017 at 18:00	13/07/2017	none	2 messages
	John Snow	18 (+5)	21/07/2017 at 16:45	30/05/2017	none	none
	Natasha Romanoff	12 (-4)	27/07/2017 at 17:30	23/06/2017	none	1 message
	Incredible Hulk	15 (+4)	Arrange session	01/07/2017	1 worksheet	none
	Nina Marquez	8 (-)	Arrange session	15/07/2017	2 worksheets	5 messages
	Roberto Gonzalez	14 (-3)	Arrange session	02/06/2017	none	none

View my past clients

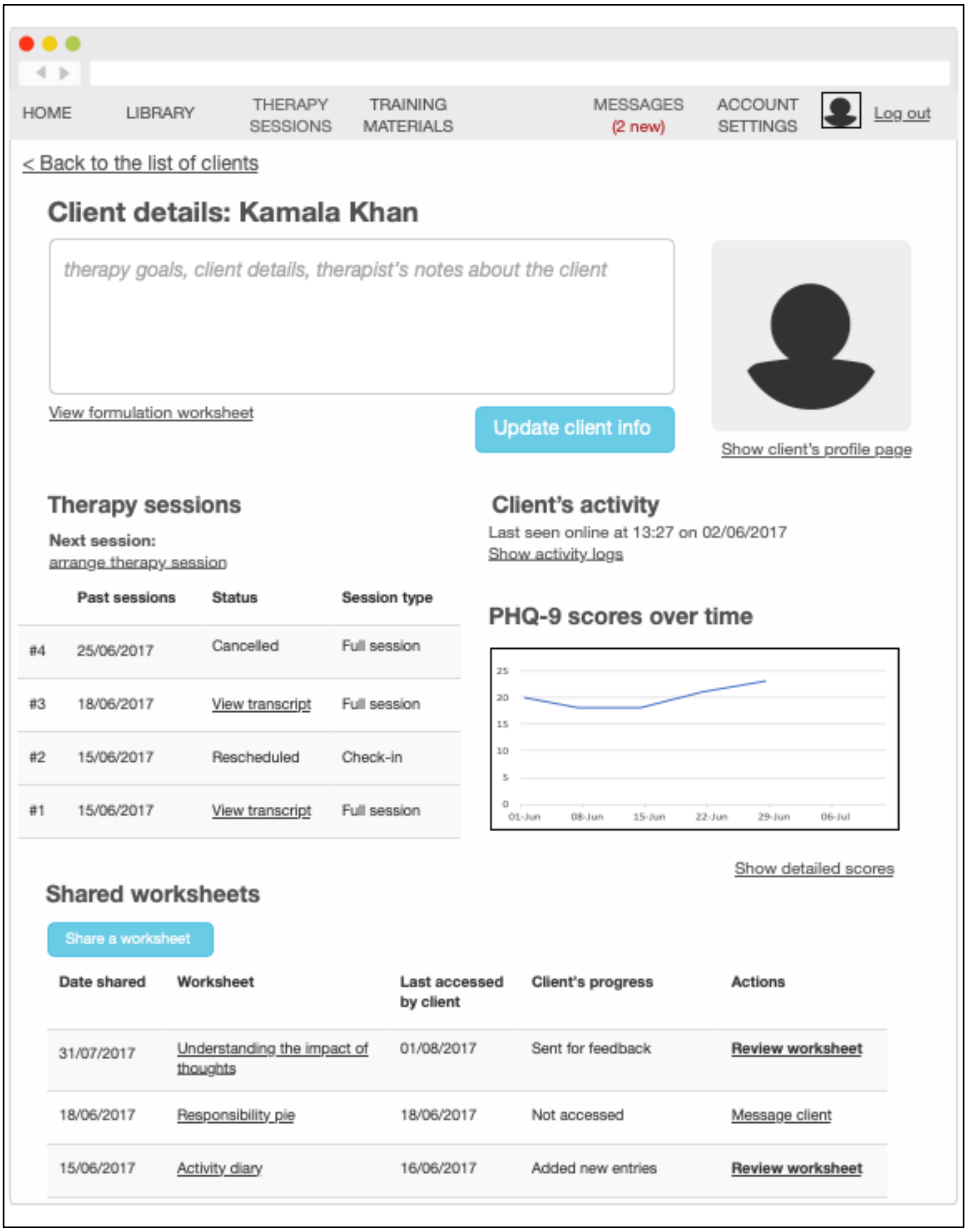
HOME
LIBRARY
THERAPY SESSIONS
TRAINING MATERIALS
MESSAGES (2 new)
ACCOUNT SETTINGS
Log out
< Back to the list of clients
Client details: Kamala Khan
therapy goals, client details, therapist's notes about the client
View formulation worksheet
Update client info
Show client's profile page
Therapy sessions
Next session:
arrange therapy session
Past sessions
Status
Session type
#4 25/06/2017 Cancelled Full session
#3 18/06/2017 View transcript Full session
#2 15/06/2017 Rescheduled Check-in
#1 15/06/2017 View transcript Full session
Client's activity
Last seen online at 13:27 on 02/06/2017
Show activity logs
PHQ-9 scores over time
25
20
15
10
5
0
01-Jun
08-Jun
15-Jun
22-Jun
29-Jun
06-Jul
Show detailed scores
Shared worksheets
Share a worksheet
Date shared
Worksheet
Last accessed by client
Client's progress
Actions
31/07/2017 Understanding the impact of thoughts 01/08/2017 Sent for feedback Review worksheet
18/06/2017 Responsibility pie 18/06/2017 Not accessed Message client
15/06/2017 Activity diary 16/06/2017 Added new entries Review worksheet
