# Supplementary material for: Design Considerations for the Integrated Delivery of Cognitive Behavioral Therapy for Depression: User-Centered Design Study
Source: JMIR Ment Health. 2020 Sep 3;7(9):e15972. doi: 10.2196/15972 (PMC7499168; doi:10.2196/15972)
Supplement: Multimedia Appendix 6 [file mental_v7i9e15972_app6.pdf]

Appendix 6. Study materials used during role-plays (Study 4)

Session script

1. Patient's first login (both therapist participant's do this activity together)

1. Log in as a patient 'Alicia'
2. Describe the patient's home page
3. Prepare for the first therapy session
    a. Update therapy goals *[give user persona]*
    b. Explore the Library
4. Log out

2. Therapist's first login and face-to-face session (both therapist participant's do this activity together)

1. Log in as Carol Danvers (therapist) and describe the home page
2. Learn more about Alicia
3. Schedule a face-to-face session to start in 60 minutes
4. Log out

3. Preparations for the face-to-face session

1. Log in as Alicia (patient) again
2. Review session preparation links on patient's home page

4. Preparations for an online session

[Therapist A logs in as therapist 'Peter Quil'l, Therapist B (or a researcher) logs in as patient 'Mandy']

[Give Mandy's persona to both therapists; give Mandy's profile to the therapist who is role-playing her]

1. Peter (therapist): schedule a session to start in 60 minutes
2. Mandy (patient): fill in the agenda and PHQ-9
3. In the meantime, Peter (therapist):
    a. Describe the home page
    b. Describe the therapy sessions page
4. When Mandy (patient) is ready:
    a. Peter (therapist): review the information sent by Mandy
    b. Peter (therapist): select worksheets that may be useful for this session

5. Online session

1. Peter (therapist) and Mandy (patient):
    a. Open the session screen

b. Describe the session screen

2. Start the session [give Peter (therapist) the task list]

6. Complete the SUS questionnaire.

7. General discussion about the platform and the role-play

Patient personas

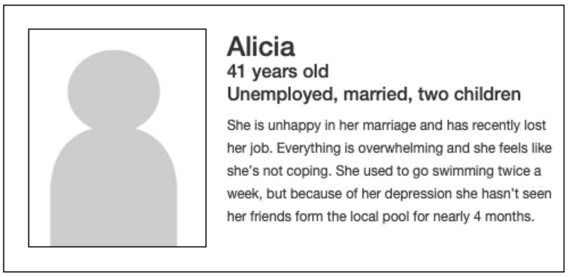

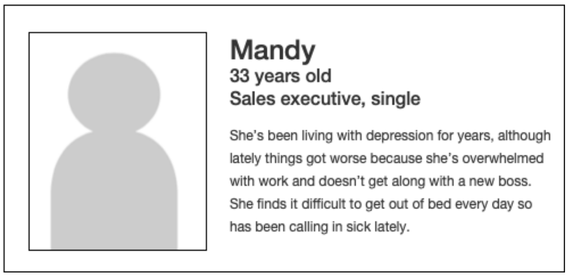

NB. The personas used during workshops had pictures representing real people. However, while the photo license allowed use to them during research, we were unable to use them as part of this publication and had to replace them with placeholders.

Patient profile

Background:
Mandy's manager joined their company a few weeks ago. During his first week, she was late a few meetings with him because she finds it difficult to get out of bed before depression. He wasn't happy about it, but she didn't mention her problems. Overall, she's struggling with depression but she doesn't want to talk about it to anyone at work.

What happened since the last session:
Last week she didn't finish writing a report on time and missed a deadline. Then, a couple of days later, her boss asked her to schedule a 1-on-1 meeting with him, but didn't say why. She called in sick the day after because she's worried he's going to fire her. Mandy hasn't been in the office since then and spent the last few days in bed.

Mandy's Therapy Aims
What do you want help with?
I need help with my depression. I can't focus on work and I can't get out of bed. This causes me stress at work.

What do you want to be different after therapy?
I want to be able to manage my work better and get up from bed without this being a terrible struggle. I don't want to lose my job

Task list

For the therapist: During the therapy session, try to pretend it is a real session. At the same time, please try to cover the following:

- Work together with the client on a worksheet
- Agree and set up homework tasks

- Write session notes together

SUS questionnaire

Please think about the platform you've been using during this session and check the box that reflects your immediate response to each statement.

1. I think that I would like to use this platform frequently.

Strongly disagree	1	2	3	4	5	Strongly agree

2. I found it unnecessarily complex.

Strongly disagree	1	2	3	4	5	Strongly agree

3. I thought it was easy to use.

Strongly disagree	1	2	3	4	5	Strongly agree

4. I think that I would need the support of a technical person to be able to use it.

Strongly disagree	1	2	3	4	5	Strongly agree

5. I found the various functions in the platform were well integrated.

Strongly disagree	1	2	3	4	5	Strongly agree

6. I thought there was too much inconsistency.

Strongly disagree	1	2	3	4	5	Strongly agree

7. I imagine that most people would learn to use this platform very quickly.

Strongly disagree	1	2	3	4	5	Strongly agree

8. I found it very awkward to use.

Strongly disagree	1	2	3	4	5	Strongly agree

9. I felt very confident using the platform.

Strongly disagree	1	2	3	4	5	Strongly agree

10. I needed to learn a lot of things before I could get going with this platform.

Strongly disagree	1	2	3	4	5	Strongly agree

Prototype screenshots

The screenshots were extracted from screen capture recordings taken during the role-play sessions to illustrate some parts of the platform used during the study. All interactions on the screen were recorded using the Silverback app (https://silverbackapp.com/) and its logo appears in the corner of the screenshots. All names and patient information on the screenshots is fictitious.

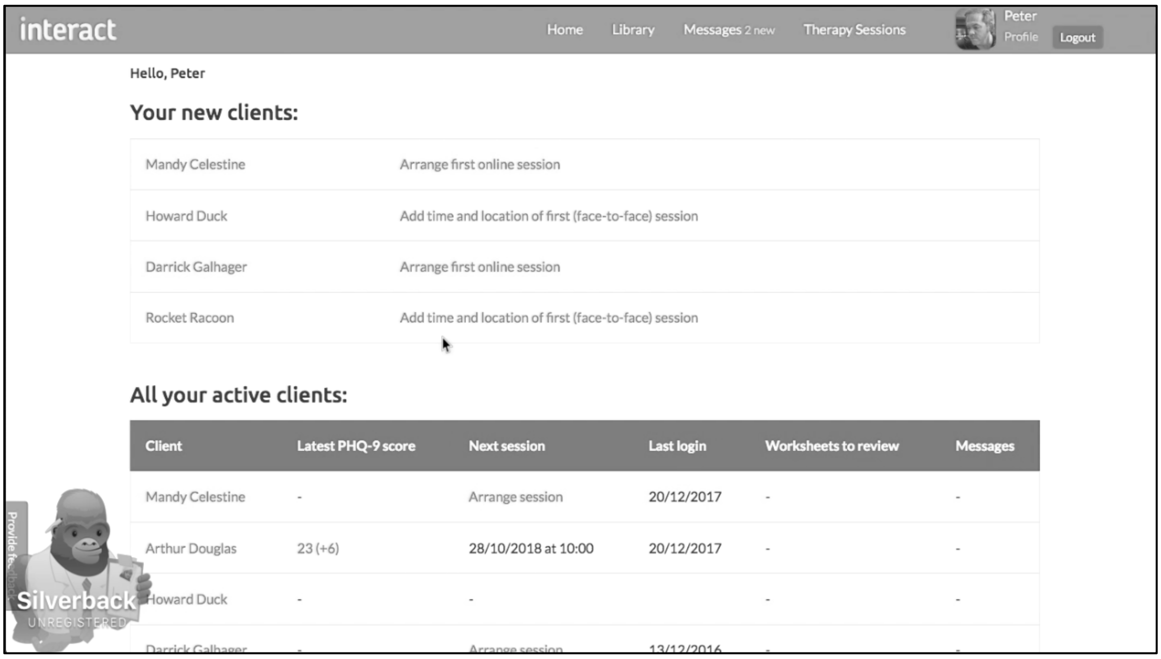

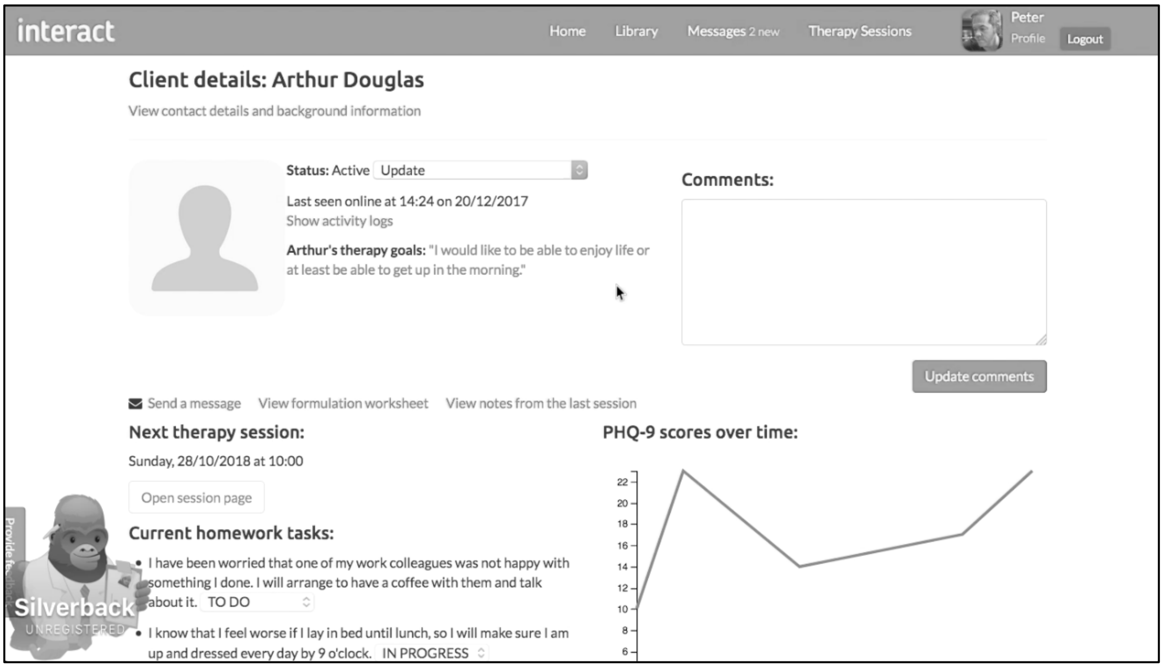

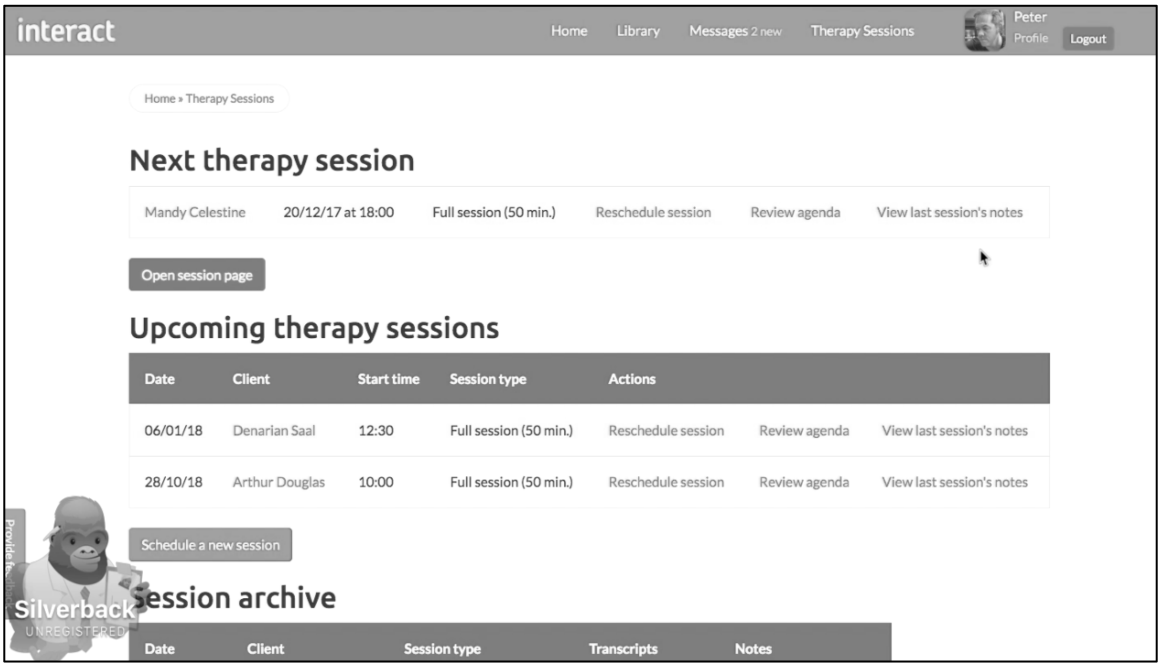
interact
Home
Library
Messages 2 new
Therapy Sessions
Peter
Profile
Logout
Home » Therapy Sessions
Next therapy session
Mandy Celestine
20/12/17 at 18:00
Full session (50 min.)
Reschedule session
Review agenda
View last session's notes
Open session page
Upcoming therapy sessions
Date
Client
Start time
Session type
Actions
06/01/18
Denarian Saal
12:30
Full session (50 min.)
Reschedule session
Review agenda
View last session's notes
28/10/18
Arthur Douglas
10:00
Full session (50 min.)
Reschedule session
Review agenda
View last session's notes
Schedule a new session
Session archive
Date
Client
Session type
Transcripts
Notes
Silverback
UNREGISTERED

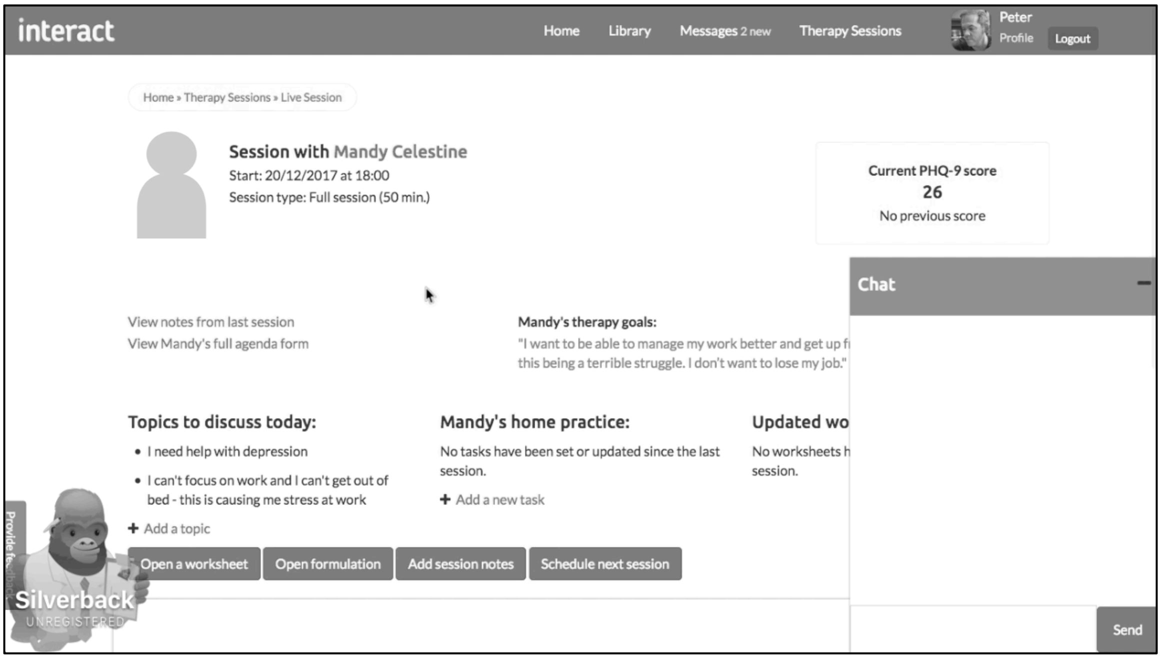
interact
Home
Library
Messages 2 new
Therapy Sessions
Peter
Profile
Logout
Home » Therapy Sessions » Live Session
Session with Mandy Celestine
Start: 20/12/2017 at 18:00
Session type: Full session (50 min.)
Current PHQ-9 score
26
No previous score
Chat
View notes from last session
View Mandy's full agenda form
Mandy's therapy goals:
"I want to be able to manage my work better and get up f
this being a terrible struggle. I don't want to lose my job."
Topics to discuss today:
I need help with depression
I can't focus on work and I can't get out of bed - this is causing me stress at work
Add a topic
Mandy's home practice:
No tasks have been set or updated since the last session.
Add a new task
Updated wo
No worksheets h
session.
Open a worksheet
Open formulation
Add session notes
Schedule next session
Send
Silverback
UNREGISTERED
